# The Molecular Basis of Depression: Implications of Sex-Related Differences in Epigenetic Regulation

**DOI:** 10.3389/fnmol.2021.708004

**Published:** 2021-07-01

**Authors:** Ayako Kawatake-Kuno, Toshiya Murai, Shusaku Uchida

**Affiliations:** ^1^SK Project, Medical Innovation Center, Kyoto University Graduate School of Medicine, Kyoto, Japan; ^2^Department of Psychiatry, Kyoto University Graduate School of Medicine, Kyoto, Japan

**Keywords:** epigenetics, stress, depression, resilience, sex differences, neural plasticity

## Abstract

Major depressive disorder (MDD) is a leading cause of disability worldwide. Although the etiology and pathophysiology of MDD remain poorly understood, aberrant neuroplasticity mediated by the epigenetic dysregulation of gene expression within the brain, which may occur due to genetic and environmental factors, may increase the risk of this disorder. Evidence has also been reported for sex-related differences in the pathophysiology of MDD, with female patients showing a greater severity of symptoms, higher degree of functional impairment, and more atypical depressive symptoms. Males and females also differ in their responsiveness to antidepressants. These clinical findings suggest that sex-dependent molecular and neural mechanisms may underlie the development of depression and the actions of antidepressant medications. This review discusses recent advances regarding the role of epigenetics in stress and depression. The first section presents a brief introduction of the basic mechanisms of epigenetic regulation, including histone modifications, DNA methylation, and non-coding RNAs. The second section reviews their contributions to neural plasticity, the risk of depression, and resilience against depression, with a particular focus on epigenetic modulators that have causal relationships with stress and depression in both clinical and animal studies. The third section highlights studies exploring sex-dependent epigenetic alterations associated with susceptibility to stress and depression. Finally, we discuss future directions to understand the etiology and pathophysiology of MDD, which would contribute to optimized and personalized therapy.

## Introduction

**Figure 1:**
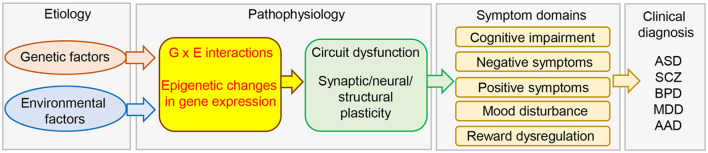
Schematic diagram of the etiology and pathophysiology of psychiatric disorders. Genetic and environmental factors and their interactions modulate brain gene expression in the brain *via* epigenetic mechanisms. This would lead to circuit dysfunction caused by aberrant synaptic, neural, and structural plasticity, and induce the expression of a variety of symptoms. AAD, alcohol abuse; ASD, autism spectrum disorder; BPD, bipolar disorder; MDD, major depression; SCZ, schizophrenia.

Depression is a heterogeneous and multifactorial disorder with a very high prevalence worldwide and is the single most important risk factor for suicide (Turecki, 2014). A meta-analysis of a large body of genetic epidemiological studies indicated that the heritability of major depressive disorder (MDD) is between 31% and 42%, which is relatively low compared to that of schizophrenia and bipolar disorder (approximately 80% and 85%, respectively). This suggests that factors other than genetic factors also contribute to the risk of depression. Indeed, human genome-wide association studies (GWAS) have largely failed to produce reproducible gene loci that contribute significantly to MDD. Although a recent GWAS identified more than 100 independent loci that are significantly associated with depression, many of which are related to synaptic structure and function (Howard et al., 2019; Ormel et al., 2019), such studies only indicate associations between genetic loci and traits and do not provide insight into causal links to underlying biological mechanisms. Moreover, the effect sizes of individual genetic variants are very small (Ormel et al., 2019). While genetic factors are important, environmental factors, such as stress, are also known to contribute to the etiology of this complex psychiatric disorder (Russo et al., 2012; Southwick and Charney, 2012). As stressful life events are known to be associated with a high risk of MDD (Hammen, 2005), the current working hypothesis is that highly complex genetic differences and environmental factors work together to determine resilience and susceptibility to MDD (Russo et al., 2012; Sun et al., 2013; Nestler, 2014; Uchida et al., 2018; Figure 1).

There is accumulating evidence for macroscopic and microscopic brain alteration in patients with MDD. Large numbers of postmortem and neuroimaging studies have reported reductions in gray matter volume and glial density in the prefrontal cortex (PFC) and the hippocampus of MDD patients, which are related to the cognitive and emotional aspects of depression, including feelings of worthlessness and guilt (Drevets, 2001; Harrison, 2002). In addition, functional neuroimaging studies using functional magnetic resonance imaging (fMRI) or positron-emission tomography (PET) demonstrate chronic increases in the activity within the amygdala and subgenual cingulate cortex (Cg25, a subregion of the PFC) in depressed individuals (Mayberg et al., 1999; Drevets, 2001; Furmark et al., 2002; Siegle et al., 2006). An electron microscopy study demonstrated reduced numbers of dendritic spines within the dorsolateral PFC of patients with MDD (Kang et al., 2012). Preclinical studies also suggested that environmental factors, such as stress, have adverse effects on the structural and functional plasticity of the nervous system (Flavell and Greenberg, 2008; Greer and Greenberg, 2008; Maze et al., 2013). For example, chronic stress was shown to induce shrinkage of dendrites of hippocampal CA3 and dentate gyrus neurons as well as loss of spines in CA1 neurons (McEwen, 1999). Other studies indicated reductions in dendritic spine density in the hippocampus and prefrontal cortex in animal models of depression (Abe-Higuchi et al., 2016; Higuchi et al., 2016; Nie et al., 2018; Moda-Sava et al., 2019). These preclinical studies together with human studies suggest that dysregulation of neuronal plasticity caused by chronic stressful life events may contribute to the pathophysiology of MDD.

Exposure to stressful environments has been shown to play a significant role in sustained alterations in gene expression (Tsankova et al., 2007; Nestler, 2014; Uchida et al., 2018). The term epigenetics refers to changes in gene expression without underlying DNA sequence alterations. These changes are heritable but environmentally modifiable (Jaenisch and Bird, 2003). Epigenetic regulation of gene expression plays fundamental roles in cellular function, neuroplasticity, and behavior (Borrelli et al., 2008), and has been suggested to be associated with not only physiological processes but also pathological conditions (Figure 1). In fact, both clinical and preclinical studies have shown that epigenetics plays an important role in the etiology and pathophysiology of depression.

Females are two to three times more likely to develop depression (Kessler et al., 2005; Whiteford et al., 2013; Malhi and Mann, 2018) and exhibit greater symptom severity, greater functional impairment, and more atypical depressive symptoms than males (Kessler et al., 1993; Kornstein et al., 2000b). Furthermore, there are differences in response to antidepressant treatment between males and females with MDD (Kornstein et al., 2000a; Khan et al., 2005). Therefore, it is necessary to understand the mechanisms underlying these sex-related differences to gain insight into the etiology and pathophysiology of depression.

Here, we review the current consensus regarding the roles of epigenetic regulation in neuronal plasticity, behavioral responses to stress, and depression, as well as their implications for the sex-related differences in the etiology and pathophysiology of depression. We focus mainly on epigenetic modulators that have been suggested to play roles in the response to stress and depression in both clinical and preclinical studies.

## Epigenetic Modifications

**Figure 2:**
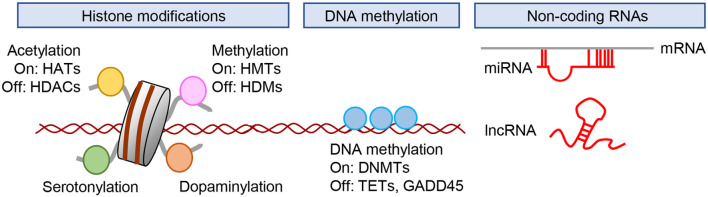
Epigenetic regulation of gene expression. Gene expression is regulated by posttranslational modification of histones, DNA methylation, and the actions of non-coding RNAs. DNMTs; DNA methyltransferases; HATs, histone acetyltransferases; HDACs, histone deacetylases; HDMs, histone demethylases; HMTs, histone methyltransferases; lncRNA, long non-coding RNA.; miRNA, microRNA; TETs, ten-eleven translocation.

The definition of epigenetics has changed over time, and the term is now used to refer to the potentially heritable but environmentally modifiable regulation of genetic function and expression. These alternations are mediated mainly through histone acetylation and methylation, DNA methylation, and mechanisms that are not encoded in the DNA (Kouzarides, 2007; Suzuki and Bird, 2008; Sun et al., 2013; Uchida et al., 2018). Here, we present a brief summary of major epigenetic modifications, including histone acetylation, histone methylation, DNA methylation, and non-coding RNAs (Figure 2).

### Histone Modifications

Histone acetylation is associated with the regulation of gene expression. Acetylation at lysine residues negates the positive charges of lysine residues in histone tails and increases the spacing between nucleosomes. This results in increased gene expression due to the decondensation of chromatin and enhanced accessibility of transcription factors to promoters (Kouzarides, 2007). The enzymes that add acetyl groups to histone tails are called histone acetyltransferases (HATs), whereas those that remove acetyl moieties from lysine residues are called histone deacetylases (HDACs; Figure 2).

Histone methylation at lysine residues has been reported in depression. A lysine residue can be mono-, di-, or trimethylated. Lysine methylation is catalyzed by a group of lysine methyltransferases (KMT) and is reversed by histone lysine demethylases (Figure 2). Site- and state-specific methylation have different functional effects possibly mediated by the recruitment of different complexes of proteins with reader domains that specifically recognize these various modifications (Sun et al., 2013). Among the five residues in the N-terminal tails of histones H3 and H4 (H3K4, H3K9, H3K27, H3K36, and H4K20), H3K9, H3K27, and H4K20 methylation are related to transcriptional silencing and heterochromatin formation/maintenance, whereas H3K4 and H3K36 methylation activate transcription (Sun et al., 2013).

In addition to histone acetylation and methylation, there are many other types of histone modifications, including phosphorylation, adenosine diphosphate (ADP) ribosylation, ubiquitination, and SUMOylation. Given that the roles of these histone modifications in stress and depression are less well understood, this review mainly focuses on histone acetylation and methylation.

### DNA Methylation

DNA methylation involves the covalent addition of a methyl group to the C5 position of cytosine (5mC) predominantly at cytosine-guanine dinucleotides (CpG sites; Suzuki and Bird, 2008). CpG islands are genomic regions with high CpG density where methylation occurs frequently, and are located in nearly 60% of promoters of protein-coding genes in the human genome (Dworkin et al., 2009). DNA methylation within gene promoters generally prevents the association of DNA binding factors with their target sequences or binding to methyl-CpG binding proteins to recruit transcriptional corepressors and exerts a repressive effect on gene transcription (Lister et al., 2013). Methylation of DNA within the promoter regions of gene plays an important role in cell differentiation, imprinting, and X chromosome inactivation (Barakat and Gribnau, 2012; Nishiyama et al., 2013; Inoue et al., 2017). Methyl-CpG- binding protein 2 (MeCp2) is a member of the methyl-CpG binding proteins, and is capable of binding specifically to methylated DNA, thereby recruiting HDACs to induce chromatin condensation and/or block transcription factor binding, both of which reduce gene expression. In addition to its role in the repression of transcription, DNA methylation is also involved in transcription activation by the recruitment of MeCP2 and transcription coactivators, such as CBP (Chahrour et al., 2008; Uchida et al., 2011). DNA methylation is catalyzed by DNA methyltransferases (DNMTs; Figure 2), while several candidate demethylases have been suggested to affect DNA demethylation. These include members of the ten-eleven translocation (TET) proteins, which oxidize 5mC to 5-hydroxymethylcytosine (5hmC) and subsequently to 5-formylcytosine and 5-carboxylcytosine. In contrast to the generally repressive effect of 5mC on gene expression, 5hmC is correlated more with transactivation (Nestler et al., 2016).

### Non-coding RNAs

Non-coding RNAs (ncRNAs) are thought to play key roles in regulating gene expression, RNA splicing, and RNA editing to determine how the genotype gives rise to the phenotype (Sumazin et al., 2011). ncRNAs are abundantly expressed in the brain in a region- and cell-type specific manner (Lau et al., 2008; Webb et al., 2015). MicroRNAs (miRNAs) are small (generally <200 bp) non-coding RNAs that play important roles in the post-transcriptional regulation of mRNA (Figure 2). They regulate the translation of mRNAs by binding to the 3′ untranslated region (UTR) or 5′ UTR of mRNAs in a sequence-specific manner (Kosik, 2006). Roles of miRNAs have been suggested in various aspects of neuronal function, including neurogenesis, synaptic plasticity, and behavior. Long non-coding RNAs (lncRNAs) are a class of RNA molecules >200 bp in length with low protein-coding potential (Qureshi and Mehler, 2012) that have been identified as important regulators of chromatin modification, miRNA sequestration, mRNA transcription, and splicing as well as acting as protein scaffolds (Wang and Chang, 2011). Although many types of ncRNA have been identified (Cech and Steitz, 2014), this review will briefly cover miRNAs and lncRNAs and their roles in neural plasticity and behaviors, as accumulating evidence suggests that these ncRNAs may be involved in the pathophysiology of stress-related psychiatric disorders.

## Epigenetics in Neuroplasticity and Depression

Changes in gene expression are required for long-term neuronal plasticity in the brain (MccLung and Nestler, 2008; Cholewa-Waclaw et al., 2016). Gene expression is controlled by a series of DNA binding proteins known as transcription factors, one of the best characterized of which is the cAMP response element-binding protein (CREB) that binds to the cAMP response element (CRE) in many gene promoters, including growth factors, enzymes, structural proteins, and other transcription factors (Lonze and Ginty, 2002). Alterations in CREB activity and expression levels are associated with the pathophysiology of depression and the efficacy of treatments (Conti et al., 2002; Manners et al., 2019). CREB activity is downregulated in the hippocampus by stress, leading to a reduction of brain-derived neurotrophic factor (*Bdnf*) mRNA (Nestler et al., 2002). Chronic antidepressant administration increases the expression of *Creb* mRNA, and activation of CREB in the hippocampus can produce antidepressant behaviors (Nibuya et al., 1996; Chen et al., 2001; Blendy, 2006). These findings indicate that alterations in CREB expression are common effects of the stress response or antidepressant treatments that may lead to the regulation of the expression of plasticity-related genes, such as *Bdnf* and its receptor, *Trkb*, which has recently been reported as a common target for the antidepressant actions of imipramine, fluoxetine, and ketamine (Casarotto et al., 2021).

It should be noted that antidepressants and psychotherapy combined can be more effective for the treatment with depression than either treatment alone in humans (Pampallona et al., 2004). This was confirmed in the animal model of anxiety and fear disorders such as posttraumatic stress disorder (PTSD). Chronic antidepressant (fluoxetine) treatment increases neuroplasticity and leads to long-term loss of fearful memories through a combination of fluoxetine treatment and extinction training, whereas fluoxetine or extinction training alone did not produce long-term fear removal (Karpova et al., 2011). Its underlying mechanism is that chronic treatment with fluoxetine reactivates juvenile-like plasticity by increasing the *Bdnf* transcript in the amygdala (Karpova et al., 2011). Similar effects of antidepressant treatment on long-term synaptic plasticity were reported previously in the adult rat visual cortex (Maya Vetencourt et al., 2008). In addition, hippocampal neurogenesis is a unique form of neural circuit plasticity that is regulated by environmental factors, including chronic stress (Van Praag et al., 2000; Sahay and Hen, 2007; Wan et al., 2015), and the reactivation of developmental plasticity also occurred in this brain structure during adulthood. The chronic antidepressant treatment induces dematuration of neurons in the dentate gyrus of the hippocampus (Kobayashi et al., 2010). Thus, the evidence suggests that plasticity-inducing treatments such as antidepressant treatment reorganize brain networks rendered more plastic by the drug treatment (Castrén and Hen, 2013). This “network hypothesis” has recently emerged as one of the mechanisms for depression and antidepressant action. This hypothesis proposes that deficits in activity-dependent neuroplasticity and neural communication might be associated with depression, and antidepressant treatment may improve the affected neural networks (Castrén, 2005; Leistedt and Linkowski, 2013). Here, we focus on how epigenetics influences the expression of genes associated with neuroplasticity and the implications in stress and depression.

### Histone Modifications

Many studies have demonstrated that HDACs affect depression and/or the actions of antidepressants. HDAC2 was reported to be involved in neural and synaptic plasticity. Guan et al. (2009) reported that HDAC2 activation reduced the number of synapses and decreased synaptic plasticity. They also demonstrated that HDAC2 is bound to the promoters of several genes involved in synaptic plasticity, including *Bdnf*. Moreover, loss-of-function mutation of HDAC2 restores synaptic plasticity deficits and increases synapse number (Gräff et al., 2012). Thus, HDAC2 is known to play a critical role in the epigenetic control of neuronal and synaptic plasticity.

Several lines of evidence suggest a key role of HDAC2 in the response to stress and depression. A genotype × environment (GxE) animal model of depression showed increased *Hdac2* expression in the nucleus accumbens, whereas a GxE animal model of resilience showed no alterations of *Hdac2* expression (Uchida et al., 2011). Mice overexpressing HDAC2 in the nucleus accumbens exhibit more depression-like behavior (Uchida et al., 2011), and HDAC inhibitor was shown to ameliorate the depression-like behavior associated with exposure to chronic stress (Covington et al., 2009; Uchida et al., 2011). Glial cell-derived neurotrophic factor (*Gdnf*) is one of the candidate target genes for HDAC2 associated with stress and depression. Chronic stress decreases the expression of *Gdnf* *via* epigenetic regulation of HDAC2, and HDAC2 knockdown or HDAC inhibitor treatment prevents the stress-induced reduction of *Gdnf* expression. These findings suggest that chronic stress activates HDAC2 function and that this can suppress *Gdnf* transcription, thereby inducing depression-like behaviors. It should be noted that patients with MDD show increased *Hdac2* expression and reduced *Gdnf* expression in the peripheral blood cells and whole blood (Takebayashi et al., 2006; Otsuki et al., 2008; Hobara et al., 2010), supporting a contribution of the HDAC2–GDNF pathway to the depression.

HDAC4 and HDAC5 are other important molecules involved in epigenetics. HDAC4 binds to chromatin, MEF2A, and CREB, resulting in histone deacetylation and suppression of gene expression in neurons (Chen and Cepko, 2009; Uchida and Shumyatsky, 2018). Loss-of-function mutation of HDAC4 affects the transcription of genes essential for synaptic function, including *Camk2a* and *Homer1* (Sando et al., 2012). HDAC4 knockout mice show deficits in synaptic plasticity and memory performance (Kim et al., 2012). As MDD may be considered primarily as an illness of emotional/mood dysregulation, it also involves substantial cognitive dysfunction (Talarowska et al., 2016; MacQueen and Memedovich, 2017). Chronic stress increases HDAC4 expression in the hippocampus and treatment with HDAC4/5 inhibitor was shown to rescue aberrant structural plasticity and have antidepressant-like effects in mice (Higuchi et al., 2016). In addition, mice lacking HDAC5 exhibited increased depressive-like behaviors after chronic social defeat stress compared with control animals (Renthal et al., 2007). Other studies showed that HDAC5-mediated regulation of gene expression in the hippocampus is essential for the behavioral effects of chronic treatment with antidepressant drugs, such as imipramine, fluoxetine, and ketamine (Tsankova et al., 2006; Choi et al., 2015; Higuchi et al., 2016). Human studies also suggested that HDAC4 and HDAC5 are potential biomarkers for mood disorders. The levels of *HDAC5* and *HDAC4* expression in peripheral blood cells are upregulated in MDD and bipolar disorder, respectively (Iga et al., 2007; Hobara et al., 2010). Taken together, these observations support the associations of HDAC4/5 with structural and synaptic plasticity and abnormal behaviors in many neurological and psychiatric diseases.

Histone methylation has been suggested to have adaptive effects in models of stress (Covington et al., 2011; Uchida et al., 2011). Chronic stress was shown to decrease global levels of H3K9 dimethylation (H3K9me2), with coincident downregulation of the histone methyltransferase, G9a (Covington et al., 2011). Activation of G9a is involved in increased H3K9me2 in the *Camk2a* gene, which plays an important role in synaptic plasticity, and induced antidepressant effects (Covington et al., 2011). H3K4 trimethylation (H3K4me3) levels are also decreased by chronic stress (Uchida et al., 2011). As the pathway related to H3K4 methylation has been suggested to be involved in major psychiatric disorders (Akbarian and Huang, 2009; Shen et al., 2014; Network and Pathway Analysis Subgroup of Psychiatric Genomics Consortium, 2015), stress-mediated alteration of H3K4me3 may underlie common mechanisms of stress-related psychiatric symptoms.

Postmortem analysis of the brains of depressed suicide victims demonstrated enrichment of H3K9me3 in the connexin 30 and 43 genes in the prefrontal cortex, the expression of which was previously reported to be downregulated in the prefrontal cortex of depressed suicide victims (Nagy et al., 2017). Cruceanu et al. (2013) reported significantly increased levels of H3K4me3 in the *Synapsin* gene, which is related to plasticity, in patients with MDD and bipolar disorder, and this effect was correlated with significant increases in gene expression, suggesting that synapsin dysregulation in mood disorders is at least partially mediated by epigenetic mechanisms.

Recently, Farrelly et al. (2019) reported that H3 is modified by the neurotransmitter, serotonin. This is the first report of a histone modified by monoamination and provides novel insight into the role of neurotransmitter signaling in a myriad of emotions and behaviors. They also showed that H3 glutamine 5 (H3Q5) serves as the primary site of serotonylation and that H3Q5 serotonylation co-occurs with H3K4me3, which is correlated with active transcription. In addition to histone serotonylation, Lepack et al. (2020) recently reported that dopamine associates with chromatin to initiate a previously unknown form of epigenetic regulation called dopaminylation. The dopaminylation of histone is associated with aberrant expression of reward-related behavior and synaptic plasticity-related neuronal genes. These observations suggest that serotonylation and dopaminylation of histone may have an impact on the etiology of neurotransmitter-related diseases (Anastas and Shi, 2019). Further studies are required to determine whether chronic stress exposure and antidepressant drugs, including the selective serotonin reuptake inhibitors (SSRIs) or other small molecules acting on monoamines, exert their effects through direct chromatin modifications, such as histone serotonylation/dopaminylation, and whether these chromatin changes determine therapeutic efficacy.

**Figure 3:**
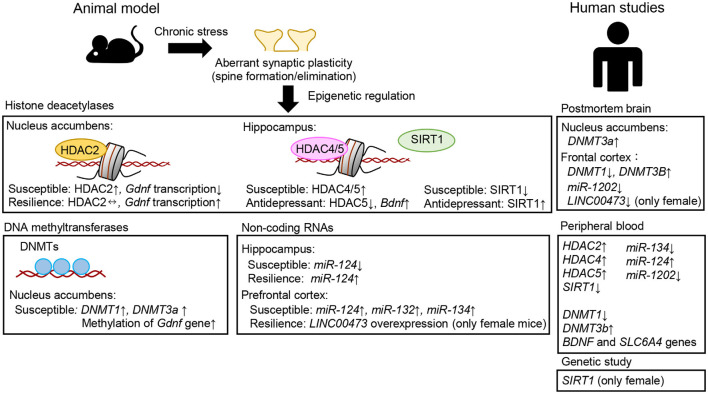
Examples of aberrant epigenetic modifications associated with neuroplasticity, stress, and depression observed in patients with major depressive disorder and/or suicide victims and in animal models of depression.

Overall, existing data indicate that histone modifications are robustly modulated by adverse environmental factors across brain regions, including the hippocampus, prefrontal cortex, and nucleus accumbens, which are brain areas known to be associated with MDD (Russo and Nestler, 2013). Accordingly, aberrant structural plasticity, such as decreased dendritic spine density in neurons, is seen in these brain areas of animal models of depression. Although the evidence presented here provides critical data showing that focal knockdown and/or overexpression of HDACs leads to alterations in gene expression, spine density, and the behavioral response to chronic stress (Figure 3), it remains unclear how histone acetylation and methylation regulate the expression of specific target genes involved in spine development, spinogenesis, or spine elimination. In addition, much effort is needed to clarify how these mechanisms are associated with depression-like behaviors and the molecular and cellular pathways that can be targeted for the treatment of depression. It should be noted that epigenetic studies will inform the development of strategies for new compounds by moving beyond monoamine transporters and receptors as targets for the treatment of depression. Indeed, as mentioned earlier, HDAC inhibitors exert potential antidepressant-like actions in several chronic stress models. Moreover, several lines of evidence have shown altered expression of HDACs in the peripheral blood cells of patients with MDD, thus highlighting the potential of HDACs to be used as biomarkers of MDD. Although genetic and environmental factors are critical to the development of depression risk and resilience, it is still unclear whether and how these factors influence individual differences in histone modifications and depression susceptibility and resilience. Thus, deciphering how genes and the environment interact at the molecular level to characterize disease risk and resilience will allow for a better understanding of the disease mechanisms and their treatment.

### DNA Methylation

The DNA methyltransferases (DNMT1, DNMT2, DNMT3A, and DNMT3B) catalyze DNA methylation. DNMT3A is responsible for the de novo establishment of DNA methylation patterns, and DNMT1 maintains the methylation pattern in a replication-coupled manner. These molecules have been shown to play important roles in neuroplasticity, depression, and antidepressant actions. A human postmortem brain study revealed decreased and increased expression levels of *DNMT1* and *DNMT3B*, respectively, in the frontopolar cortex of depressed individuals (Poulter et al., 2008). Increased *DNMT3A* expression was observed in the nucleus accumbens of individuals with MDD (Hodes et al., 2015). Moreover, in peripheral blood cells, the levels of *DNMT1* mRNA were significantly decreased in a depressive but not in a remissive state of MDD (Higuchi et al., 2011). Conversely, the levels of *DNMT3B* mRNA in MDD were significantly increased in a depressive but not in a remissive state. DNMTs are involved in the effects of stress and depression in animals. Chronic stress has been shown to increase DNMT1 and DNMT3a levels in the nucleus accumbens and treatment with DNMT inhibitors reversed depression-like behaviors (Uchida et al., 2011). Another study also indicated that DNMT regulates dendritic spine density and chronic stress-induced depression-like behaviors (LaPlant et al., 2010). Sales et al. (2011) showed that treatment with DNMT inhibitors exhibited antidepressant-like effects mediated *via* the inhibition of DNA methylation in the hippocampus and rescued stress-induced reduction of *Bdnf* expression (Tian et al., 2009). Taken together, these observations suggest that there may be an association between DNMT activity and depression.

A number of clinical studies have demonstrated associations between DNA methylation and depression. Several studies showed that DNA methylation in the promoter of the *BDNF* gene is associated with MDD. Fuchikami et al. (2011) reported that patients with MDD showed an absence of DNA methylation at certain CpG sites in exon 1 of the *BDNF* gene, and Tadić et al. (2014) reported that absence of DNA methylation in the *BDNF* gene promoter was associated with reduced response to antidepressant drugs. These observations suggest that the *BDNF* gene is related to MDD and responsiveness to antidepressants.

The neurotransmitter serotonin (5-HT) is known to play important roles in synaptic plasticity and mood/emotion, and the level of DNA methylation in the promoter of the *SLC6A4* gene, which encodes serotonin transporter (SERT), has been shown to be associated with its mRNA expression as well as depression and antidepressant responsiveness (Philibert et al., 2007; Sugawara et al., 2011; Okada et al., 2014).

As mentioned here, a growing body of evidence supports a role for DNA methylation and DNMTs in modulating the effects of chronic stress and depression. Although these studies highlight epigenetic modifications targeting specific genes associated with behavioral abnormalities, the effect of chronic stress is not restricted to a subset of candidate genes. In addition, MDD is not monogenic, but rather associated with many genes. Thus, genome-wide profiling of DNA methylation in behaviorally and/or physiologically classified animals subjected to chronic stress (Cerniauskas et al., 2019), together with the categorized MDD subgroups, will allow us to better understand the pathophysiology of this disorder. Furthermore, it is still unclear how DNA methylation-mediated regulation of changes in gene transcription influences neural plasticity and circuit homeostasis. Recently developed genome engineering tools, such as epigenome editing platforms (Yim et al., 2020) that can modulate locus-specific epigenetic modifications and subsequent gene transcription, may resolve this issue and provide novel insights into the role of locus-specific and cell-type specific epigenetic modifications in neuroplasticity and behaviors.

### MicroRNAs

Many studies have demonstrated altered expression of miRNAs in patients with psychiatric disorders, including MDD, as summarized in detail elsewhere (Allen and Dwivedi, 2020; Yoshino and Dwivedi, 2020). This review preferentially focuses on miRNAs that are known to be involved in both synaptic plasticity and depression-like behaviors in animal models, as well as those suggested to be associated with clinical depression.

Several miRNAs are involved in modulating synaptic and neuronal functions and behavior. Brain-specific miR-134 negatively regulates the size of hippocampal dendritic spines, which is mediated by inhibition of translation of the mRNA encoding a protein kinase, Limk1, which controls spine development (Schratt et al., 2006). Exposure of neurons to BDNF ameliorates this inhibition of Limk1 translation by miR-134, and may thus contribute to synaptic maturation and plasticity. In addition, miRNA-134 was reported to affect synaptic plasticity and behavior through the modulation of HDAC expression (Gao et al., 2010). Upregulation (downregulation) of SIRT1, a type III HDAC, enhances (impairs) synaptic plasticity and memory performance, and these effects are mediated *via* post-transcriptional regulation of CREB expression by miR-134. In addition, chronic stress was shown to increase miR-134 expression in the medial prefrontal cortex of rats and decrease the expression of Limk1 (Fan et al., 2018). Intracerebral infusion of miR-134-sponge into this brain region of stressed rats, which blocks miR-134 function, significantly ameliorated aberrant neuronal structures, biochemical changes, and depression-like behaviors. Another study showed that miR-124 overexpression enhanced dendritic spine density in the hippocampus under conditions of stress, whereas miR-124 inhibitor reduced dendritic spine density (Higuchi et al., 2016). Behaviorally, miR-124 overexpression drives stress resilience in mice. These changes in spine density and depression-like behaviors are mediated in part by translational inhibition of HDAC4/5 (Higuchi et al., 2016). These observations suggest that miRNAs (e.g., miR-124, miR-134) act as functional links to chromatin modification by modulating translational control of HDACs, thereby modulating neural plasticity and higher brain function. It should be noted that both miR-124 and miR-134 are associated with MDD (He et al., 2016; Roy et al., 2017; Fang et al., 2018; Zhang et al., 2020). Therefore, miR-124- and miR-134-mediated translational control may be involved in the pathophysiology of depression and the actions of antidepressants.

SERT is the pharmacological target of antidepressant drugs and is a target of miR-16 (Baudry et al., 2010). miR-16 is enriched in noradrenergic cells compared to serotonergic cells. Chronic treatment with the antidepressant drug fluoxetine increases the miR-16 expression in the serotonergic raphe nuclei, thus reducing SERT expression. In contrast, fluoxetine treatment decreases the miR-16 level and upregulates SERT expression in the noradrenergic locus coeruleus. Thus, the miR-16-SERT pathway is associated with behavioral responses to antidepressant drugs. A postmortem study showed decreased miR-16 expression in the dorsal raphe of suicide victims (Issler et al., 2014). These observations suggest that miR-16 may contribute to the therapeutic action of fluoxetine in depression and anxiety disorders.

Issler et al. (2014) reported that miR-135 is essential for stress resilience and efficacy of antidepressants. The expression of miR-135 in the dorsal raphe was shown to be upregulated by chronic treatment with the antidepressants, imipramine and fluoxetine. Overexpression of miR-135 in serotonergic neurons resulted in behavioral resilience to chronic stress, possibly due to inhibition of *SERT* and *Htr1a* mRNA translation, while miR-135 knockdown increased anxiety behavior and attenuated antidepressant responsiveness in mice. In addition, the expression of miR-135 was shown to be downregulated in patients with depression. Taken together, these results suggest that miR-135 is an essential regulatory element responsible for the responses to both stress and antidepressants.

With regard to synaptic plasticity, miRNAs may directly regulate synaptic function, as some miRNAs and their processing enzymes (*e.g*., Dicer, Drosha) were shown to be highly enriched at synaptic sites (Lugli et al., 2005, 2008; Yoshino et al., 2021). Importantly, neural activity facilitated local protein translation by releasing miRNAs from certain mRNAs (Bicker et al., 2013; Hu et al., 2014). Yoshino et al. (2021) reported that a large number of miRNAs are enriched in synapses and that patients with MDD showed altered expression of synaptic miRNAs, thus providing new insights into the roles of synaptic miRNAs and suggesting their involvement in the pathogenesis of MDD. However, there have been few studies regarding the roles of synaptic miRNAs and their involvement in neuropsychiatric disorders.

There have also been reports regarding the relations of miRNAs with antidepressant treatment response. The miRNAs miR-46a/b-5, miR-425–3p, and miR-24–3p show alterations in their expression levels in response to chronic antidepressants, which are known to regulate MAPK/Wnt-system genes (Lopez et al., 2017). miR-598-5p has been shown to be a common target of three treatments for depression, i.e., antidepressant (fluoxetine), ketamine, and electroconvulsive therapy (O’Connor et al., 2013). Moreover, Lopez et al. (2014) reported that miR-1202 dysregulation detected in the postmortem brain and peripheral blood was associated with the pathophysiology of MDD. They postulated that miR-1202 may be a useful biomarker of MDD and a predictor of antidepressant treatment response. Thus, these studies highlight the roles of miRNAs in MDD and may facilitate the development of novel methods for effective pharmacological treatment of mood disorders.

Early life stress is a potent neurodevelopmental disruptor and increases the risk for MDD (Heim and Nemeroff, 2001; Park et al., 2019). Early life stress leads to hypothalamic-pituitary-adrenal (HPA) axis impairment and immune reactions, which increase vulnerability to MDD and suicidal behavior (Fagundes et al., 2013; Allen and Dwivedi, 2020; Juruena et al., 2020). There is accumulating evidence that the underlying mechanisms involve epigenetic modifications by miRNAs. For example, although maternal separation enhances the risk of a depression-like phenotype in adulthood, this early life stress was shown to increase miR-504 expression in the nucleus accumbens and miR-132, miR-124, miR-9, and miR-29a expression in the medial prefrontal cortex of rodents (Uchida et al., 2010; Zhang et al., 2013). Interestingly, in a study investigating the relations between miR-9 levels in blood samples, fMRI data, and experience of childhood maltreatment in human MDD patients, He et al. (2021) reported that machine learning has the potential to allow differentiation of MDD patients from healthy controls by integrating miR-9 levels, the severity of childhood maltreatment, and strength of amygdala functional connectivity in the prefrontal-limbic regions.

To date, there is accumulating evidence for the involvement of brain miRNAs in the epigenetic effects of chronic stress and the development of stress-related psychopathologies. However, there is a need for a multiscale analysis to decipher how miRNA-mediated epigenetic regulation influences spine development, neuroplasticity, and behaviors. As mentioned earlier, miRNAs are localized at synaptic sites, but their functions are still unknown. In addition, many miRNAs are expressed in a cell-type-specific manner, and nearly all studies to date have assessed miRNA function in heterogeneous populations of cells. The recent advancements in technology, including cell type- and circuit-specific viral-mediated gene transfer, epigenome editing, and single-cell analysis, allow us to analyze specific cell populations *in vivo*; nevertheless, the elucidation of the epigenetic mechanisms of miRNAs in distinct cell types, including neurons, astrocytes, microglia, and oligodendrocytes, and in distinct neural circuits are required. Indeed, recent evidence has implicated epigenome/transcription changes in astrocytes and oligodendrocytes in patients with MDD (Nagy et al., 2015, 2020; Lutz et al., 2017).

### Long Non-coding RNAs

lncRNAs have emerged as key components of gene regulatory networks in concert with other key molecules, such as epigenetic modifiers and transcription factors, which are associated with brain function. Indeed, accumulating evidence suggests the important roles of lncRNAs in neuronal development and plasticity. The lncRNA BDNF-AS, which prevents *Bdnf* transcription by recruiting a key component of epigenetic silencing complex, was reported to be associated with many psychiatric disorders, including depression (Ng et al., 2013). In addition, inhibition of BDNF-AS was shown to induce the upregulation of *Bdnf* expression, leading to increased *Gdnf* expression as well as enhanced neuronal maturation (Modarresi et al., 2012), which were suggested to be associated with antidepressant action and stress resilience. Thus, BDNF-AS lncRNA may contribute to the pathophysiology of depression and/or antidepressant action by regulating transcription of *Bdnf*.

Experimental studies demonstrated altered lncRNA expression in animal models of depression. In a microarray study, Roy et al. (2018) demonstrated transcriptome-wide changes in lncRNAs in the hippocampus of male rats that showed susceptibility (learned helplessness model) or resilience (non-learned helplessness) to the development of depression. Treatment with fluoxetine was also shown to be associated with changes in lncRNA expression in animals susceptible to the development of depression (Wang et al., 2019). This study suggested that specific classes of lncRNAs with distinctive roles in modulating target gene expression may be involved in susceptibility or resilience to the development of depression. They further examined how lncRNAs are coexpressed with gene transcripts and whether specific lncRNA/mRNA modules are associated with susceptibility or resilience to stress and depression by weighted gene coexpression network analysis (WGCNA) to correlate the expression status of protein-coding transcripts with lncRNAs. Their results identified five hub mRNAs (*Tas2r116*, *Expi*, *Rnf29*, *Oprs1*, and *LOC690326*) and 20 lncRNAs that are associated with susceptibility and resilience to the development of depression. These studies suggested that lncRNA-associated networks may play crucial roles in the development of depression.

Some authors have suggested that lncRNAs may be useful as diagnostic biomarkers or therapeutic targets for depression. Indeed, recent studies have detected regulatory lncRNAs that may be involved in psychiatric disorders. For example, a global non-coding RNA expression analysis of depressed suicide victims detected 23 differentially expressed lncRNAs as well as their differentially expressed overlapping and antisense protein-coding genes in the rostral anterior cingulate cortex (Zhou et al., 2018). Notably, in this analysis, one of the top differentially expressed lncRNAs was identified as RP1–269M15.3, the expression of which in the nucleus accumbens was reported previously to be upregulated by 30 MDD-related SNPs (Zeng et al., 2017; Zhou et al., 2018). In the prefrontal cortex, RNA-Seq analysis demonstrated that expression of the lncRNA, LINC01268, was upregulated in victims of violent suicide compared to both non-suicides and victims of non-violent suicide (Punzi et al., 2019).

To examine the roles of lncRNAs in depression, Seki et al. (2019) measured the expression of 83 lncRNAs in the peripheral blood leukocytes of MDD patients. Their results showed that the expression level of one lncRNA (RMRP) was lower, while those of four lncRNAs (Y5, MER11C, PCAT1, and PCAT29) were higher in MDD patients than in healthy controls. The RMRP expression level was correlated with the severity of depression as measured by the Hamilton Depression Rating Scale. Moreover, they found reduced expression of RMRP in the blood of a widely used corticosterone-induced mouse model of depression, corroborating the observations in MDD patients. This suggests that a lower RMRP level may serve as a potential biomarker for MDD. Another study using peripheral blood cells also found negative associations of six lncRNAs with suicide risk in MDD patients (Cui et al., 2017), and these lncRNAs could be potential markers for preventing suicide in MDD patients.

Here, we have described the current knowledge regarding the roles of lncRNAs in neural plasticity and summarized the contributions of some lncRNAs to stress and depression. Transcriptome analyses have highlighted alterations in the transcriptional regulatory machinery in patients with MDD, consistent with recent findings indicating that genetic mutations and/or polymorphisms linked to psychiatric disorders fall into non-coding regions of the genome. Although these studies have provided some insight into the diverse roles of lncRNAs in depression, many unanswered questions remain. For example, mammalian cells encode about 170,000 lncRNAs (Zhao et al., 2016), but little is known about how chronic stress and/or therapeutic drugs influence their expression, localization, and function in the brain. In addition, the association of lncRNAs with (epi)genetics and specific phenotypical characteristics remains largely unknown. Further studies of the roles of lncRNAs in psychiatric disorders are required to discover new targets for the development of effective drugs to treat depression.

In this part of this review, we mainly summarized the role of epigenetic molecules in chronic stress-induced maladaptive changes in structural plasticity and behaviors. In many cases, alterations in spine density and the expression of epigenetic molecules following chronic stress lead to maladaptive and susceptible phenotypes, whereas stress-resilient animals do not show any changes. What happens in the resilient brain? Studies have demonstrated that such resilience is mediated not only by the lack of key molecular changes that occur in stress-susceptible animals to block their ability to cope with chronic stress but also by the presence of distinct molecular adaptations that occur in resilient animals to help and maintain normal behavioral function (Uchida et al., 2011; Russo et al., 2012; Sakai et al., 2021). An important finding from animal studies demonstrated that gene expression changes and chromatin modifications in the reward pathway (ventral tegmental area-nucleus accumbens) contribute to dopamine transmission and adaptive behaviors. Examples of genes and epigenetic modifications involved in resilience include HDAC2, histone acetylation, and miRNAs (Krishnan et al., 2007; Uchida et al., 2011; Dias et al., 2014), as described here. However, the factors involved in stress resilience are just beginning to be identified. It should be noted that there is a great need for human brain imaging studies to identify the brain structures and circuits that mediate stress resilience. This information could be used for reverse translational research in animals and for the development of treatment strategies (e.g., the potential for deep brain stimulation) for clinical depression.

## Sex-Related Differences in Synaptic Plasticity

There have been many studies of the effects of sex steroid hormones on sexual differentiation of the brain. Estrogens are sex steroid hormones synthesized and secreted by both sexes, although the levels are considerably higher in young reproductively mature females than in males. It has been suggested that estrogen underlies the mechanism of sex-specific brain development by regulation of neuronal transmission and neuroplasticity as well as neuroprotective effects and immune functions (Zhang et al., 2001; Raison and Miller, 2013; Marrocco and McEwen, 2016). For example, the hippocampus, which plays critical roles in both mood regulation and higher cognitive functions, shows high levels of estrogen receptor expression, and this hormone has been shown to have a profound effect on hippocampal structure and function (Li et al., 2004). Estradiol shows synapse induction within the hippocampus in females but not in males, whereas aberrant hippocampal synaptic remodeling of CA3 dendrites after chronic stress exposure was seen in males but not in females, suggesting that gonadal hormones are related to susceptibility or resilience to stress (Galea et al., 1997; Leranth et al., 2003). However, whether there is a mechanism underlying the sex-specific development of MDD remains to be clarified.

The plasticity of neuron structure, including spine density, is sex-dependent, and sex steroids have significant effects on this disparity. For example, hippocampal spine density was shown to change in response to fluctuations in ovarian steroid levels across the estrous cycle in female rats (Gould et al., 1990). Furthermore, it has been suggested that hormone-dependent modifications of the NMDA receptor can lead to sex-related differences in synaptic plasticity following exposure to stress (Hyer et al., 2018). The molecular mechanisms underlying sex-dependent differences in synaptic plasticity are also related to the effects of estrogen. Estradiol is known to drive potentiation of glutamatergic synapses in both males and females. However, the mechanisms underlying the initiation of potentiation are sex-dependent. Estrogens bind with estrogen receptors ERα, ERβ, and G protein-coupled estrogen receptor 1 (GPER1). Estradiol drives potentiation through GPER1 in females, but through ERβ in males. On the other hand, presynaptic potentiation is driven by ERα in males but by ERβ in females (Oberlander and Woolley, 2016). ERα and ERβ have been shown to activate different metabotropic glutamate receptors. ERα activation triggers mGluR1 signaling and subsequently drives CREB phosphorylation. Signaling through ERβ activates mGluR2/3 and results in downregulation of calcium-mediated CREB phosphorylation (Boulware et al., 2013). These results showed that sex-specific interactions with ERα and ERβ activate different downstream regulatory pathways, which in turn affect synaptic plasticity.

## Sex-Related Differences in Depression

Etiologically, depression is two times more prevalent in females than in males. Female depressed patients show greater severity, earlier age of onset, and increased duration of depressive episodes than male patients (Marcus et al., 2005). A meta-analysis of neuroimaging studies demonstrated sex-related differences in the effects of stress on brain structure and function. The Enhancing NeuroImaging Genetics through MetaAnalysis Consortium performed brain morphometric analyses and showed that greater severity of maltreatment is related to overall lower gray matter thickness and smaller caudate volume in female depressive patients, but a decreased thickness of the rostral anterior cingulate cortex in males (Thompson et al., 2020). Also, there is accumulating evidence for sex-specific effects of stress on synaptic and neural plasticity (Shors et al., 2004; Carvalho-Netto et al., 2011; McEwen et al., 2016). Female rodents have been shown to mount a greater neuroendocrine response to stress in comparison to males (Heck and Handa, 2019; Zuloaga et al., 2020). This response may be hormone-dependent, as higher estrogen levels are associated with greater stress responses (Viau and Meaney, 1991; Lund et al., 2006; Liu et al., 2011). Furthermore, stress events can often have the opposite effects on behavior in males and females (Bowman et al., 2001; Luine, 2002; Conrad et al., 2003; Ortiz et al., 2015; Peay et al., 2020).

The efficacy of commonly used antidepressants also differs between males and females, although there is no clear consensus; men show a better therapeutic response to tricyclic antidepressants (TCAs) than women (Kornstein et al., 2000a), whereas women respond better to SSRI treatment than men (Kornstein et al., 2000a; Khan et al., 2005). Preclinical studies have shown that female mice are more responsive to ketamine, a fast-acting antidepressant, than males (Carrier and Kabbaj, 2013; Franceschelli et al., 2015). Taken together, these observations suggest that sex-dependent molecular mechanisms underlie the differences in responsiveness to antidepressants between males and females.

There is accumulating evidence that this disparity in susceptibility to depression between males and females is related to sex-specific differences in neuronal circuitry, hormone levels, and metabolism (Li et al., 2004; Fernández-Guasti et al., 2012). Notably, downregulation of the expression of a hub gene has been shown to underlie sex-related differences in depression susceptibility. *DUSP6*, which encodes dual-specificity phosphatase-6, has been shown to mediate several brain-related functions through the inactivation of the ERK pathway (Muda et al., 1996). *DUSP6* expression was shown to be downregulated in female but not male patients with depression, and in an animal study knockdown of DUSP6 was shown to increase stress susceptibility by enhancing the excitability of a population of glutamatergic pyramidal neurons in the ventromedial prefrontal cortex *via* the activation of ERK signaling (Labonté et al., 2017). However, these factors cannot fully explain the sex-related differences in susceptibility to depression, and it is possible that X-chromosome inactivation (XCI) and epigenetic factors also play major roles in the molecular and cellular mechanisms underlying these differences.

## Epigenetic Mechanisms of Sex-Related Differences in Depression

Epigenetic mechanisms have pivotal roles in sex-related differences in gene expression and sexual differentiation of the brain (Tsai et al., 2009; Shen et al., 2015). Estrogen acts through epigenetic mechanisms in addition to rapid-acting non-genomic functions *via* second messengers relevant to sex-related differences in brain function and behavior (Kelly and Levin, 2001; Li et al., 2004). Importantly, methylation of the estrogen receptor and histone acetylation are involved in the process of masculinizing the brain (Kurian et al., 2010; Matsuda et al., 2011). When testosterone reaches the brain and is converted in part to estradiol, the nuclear receptors ERα (encoded by *ESR1*) and ERβ (encoded by *ERS2*) are activated to initiate male brain organization. CpG sites in *ESR1* and *ESR2* show sex-dependent DNA methylation levels that interact with hormones at critical periods of development, affecting downstream trajectories of brain development that differ between males and females. These epigenetic sex-related differences result in disparities between males and females in vulnerability to mental disorders in humans or to repeated social defeat stress in adulthood (Golden et al., 2013; Kim et al., 2015).

Furthermore, interactions between the hypothalamic-pituitary-gonadal (HPG) and HPA axes could lead to sex differences in the regulation of stress responsivity (Oyola and Handa, 2017). Estrogens drive HPA axis activation in females, resulting in the elevation of glucocorticoids during proestrus and estrus in rodents (Figueiredo et al., 2002). Of note, estradiol inhibits restraint-induced ACTH release but strongly enhances the sensitivity of the adrenal to ACTH, which results in a net increase in stress-induced glucocorticoid release (Ulrich-Lai et al., 2006). The paraventricular nucleus of the hypothalamus (PVN) plays an important role in the activation of the HPA axis, integrating neuronal and humoral inputs to secrete corticotropin-releasing hormone (CRH; Ferguson et al., 2008; Oyola and Handa, 2017). CRH neurons in the rat PVN express ERβ, but not ERα. *In vitro* studies have demonstrated that ERβ, but not α, strongly stimulates the *Crh* promoter, suggesting that estradiol-dependent effects in the regulation of the HPA axis may be driven by their direct action through ERβ in the CRH neurons of the PVN (Miller et al., 2004). In addition, peripheral administration of the ERβ agonist, R-DPN, blocked the increase in HPA axis reactivity and induced anxiety-like behaviors in female rats in response to the implantation of a wax pellet containing a GR agonist adjacent to the central nucleus of the amygdala (Weiser et al., 2010). Together, these results suggest a possible mechanism for the action of estradiol by inhibiting HPA axis reactivity and modulating *Crh* gene expression through ERβ. However, further studies are needed to elucidate how interactions between the HPA and HPG axes cause sex-dependent differences in stress responsivity.

The X-chromosome also plays crucial role in the development of depression and other psychiatric disorders. In female cells, most genes on one X chromosome are silenced by a mechanism known as XCI that involves packaging of the inactive X-chromosome into a transcriptionally inactive structure called heterochromatin. However, this silencing process is imperfect, requiring the silencing machinery to function throughout life, with about 15% of genes on the X chromosome “escaping” XCI and an additional 10% of genes showing variable patterns of inactivation resulting in double or various doses of the gene product in females (Carrel and Willard, 2005; Johnston et al., 2008). Overdosage of X-linked genes that have escaped XCI due to the presence of an extra X-chromosome was suggested to contribute to the development of psychiatric symptoms in both Klinefelter syndrome (XXY) and Triple X syndrome (XXX), and XCI has been shown to be an important mechanism underlying the sex-related differences in depression. Nearly all of the genes that show sex-related bias in multiple tissues are located on the X- and Y-chromosomes (Reinius et al., 2010). One of the best-studied of these genes, *Xist*, is highly sexually dimorphic, with female-specific expression, and plays a pivotal role in XCI (Sahakyan et al., 2018). In addition, several other genes, including *KDM5C, EIF2S3X, KDM6A*, and* DDX3X* , have been reported to escape XCI (Reinius et al., 2010). Overexpression of X-linked genes may be a common mechanism underlying the development of psychiatric disorders in patients with these rare genetic diseases and in the general population of female psychiatric patients with *XIST* overexpression. Among the four X-linked escapee genes stated above, *KDM5C* is especially known to be related to psychiatric disorders including X-linked syndrome, autism, and depression (Ji et al., 2015; Vallianatos and Iwase, 2015; Talebizadeh et al., 2019). Indeed, female patients with MDD show increased expression of the *XIST* gene and *KDM5C* in lymphoblastoid cells (Ji et al., 2015). In addition, a recent clinical study has reported that, in blood samples, the expression levels of KDM5C-3’UTR-lncRNA isoform were different in autistic females with XCI skewness compared with controls (Talebizadeh et al., 2019). Intriguingly, a study of human postmortem brains supported overexpression of the *XIST* gene in female psychiatric patients (Ji et al., 2015).

### Sex-Related Differences in Histone Modifications and DNA Methylation in Depression and Stress

One of the first SNPs identified as related to depression by GWAS was located in the *SIRT1* gene (Ledford, 2015), which encodes a type III HDAC. The authors reported a significant association of this gene with MDD only in females. In addition, this gene was reported to be associated with MDD and female suicide victims in a Japanese population (Kishi et al., 2010; Hirata et al., 2019). Moreover, the level of *SIRT1* mRNA in peripheral blood was shown to be reduced in MDD patients compared to healthy subjects (Abe et al., 2011; Luo and Zhang, 2016). An animal study suggested that SIRT1 contributes to the development of depression (Abe-Higuchi et al., 2016). Chronic stress was shown to reduce SIRT1 expression in the hippocampus, and this effect was rescued by chronic treatment with antidepressants. Hippocampal SIRT1 activation reversed stress-induced aberrant dendritic spine density and increased depression-like behaviors, whereas hippocampal SIRT1 inhibition induced reduction of spine number and increased depression-like behaviors. These observations indicated that the function of SIRT1 in the hippocampus drives stress resilience. Although the sex-dependent role of SIRT1 in depression is still unclear, taken together with the results of clinical studies, these observations suggest that SIRT1 may be associated with the etiopathogenesis of sex-related differences in depression.

Stress events trigger the development of PTSD, and women are more than twice as likely to develop PTSD than men, similar to the case with depression (Breslau et al., 1998). The molecular mechanisms underlying the increased risk of PTSD in females are unclear, but Maddox et al. (2018) suggested that DNA methylation and HDAC4 regulation by estrogen may be associated with an increased risk of PTSD in some women. Importantly, they found that genetic and epigenetic variation can alter *HDAC4* expression, leading to PSTD in a sex-dependent manner. Their results indicated that higher methylation status at specific CpG sites and the genotype of the HDAC4 gene could be associated with lower levels of HDAC4 expression, which are associated with increased resting-state functional connectivity between areas of the brain implicated in fear expression as well as heightened fear load, particular in patients with PTSD.

Early life stress affects the HPA axis and may induce susceptibility to stress, but those effects differ between males and females through epigenetic mechanisms. For example, maternal separation was shown to result in higher exon methylation and lower expression of *Bdnf*, a gene associated with stress signaling pathways, in adulthood (Dandi et al., 2018). These effects on gene expression are significantly greater in females than in males (Coley et al., 2019). More recent studies showed that chronic stress also produces sex-specific epigenetic modifications. Chronic variable mild stress was demonstrated to significantly increase methylation of the CRH gene, which is related to the HPA axis, in females but not in males (Sterrenburg et al., 2011). In addition, these effects were specific to females at two particular CpG sites within the promoter region (Sterrenburg et al., 2011).

Some studies have demonstrated the sex differences in DNA methylation signatures in humans. Adult male suicide victims with a history of childhood abuse were shown to have NR3C1 hypermethylation compared to suicide victims without a history of abuse or non-suicidal postmortem controls (McGowan et al., 2009). Importantly, NR3C1 methylation shows a sex-dependent association between maternal depression and symptoms of anxiety and depression in children. Anxiety, depression, and behavioral symptoms in children can be predicted by prenatal stressors, maternal depression, and anxiety, and low birth weight (Hill et al., 2019). Increased NR3C1 methylation levels were only seen in girls when their mothers had reported lower maternal depression scores during pregnancy, while no evidence of such a relation was observed in boys (Hill et al., 2019). These observations suggest that prenatal stressors, including maternal depression, produce different epigenetic and early behavioral outcomes in males and females. Another study demonstrated a sex-specific cross-tissue methylation signature in the promoter region of the *SLC6A4* gene encoding serotonin transporter, which is known to be a target of antidepressants (Palma-Gudiel et al., 2019). This study showed that the levels of methylation at five contiguous CpG sites were markedly increased in peripheral blood cells and postmortem brain tissue of women compared to men.

The function of DNMT may be associated with depression, and there is evidence that this gene may also be associated with sex-related differences in stress susceptibility. Female mice are more vulnerable to stress than male mice, and females have higher levels of *Dnmt3a* expression than males (Hodes et al., 2015). Loss-of-function mutation of Dnmt3a resulted in greater resistance to stress in female mice, whereas its overexpression was associated with higher susceptibility to stress in male mice, suggesting a role of this gene in stress resistance. Thus, DNA methylation by DNMTs may be associated with sex-related differences in the development of stress-induced depression.

Recent studies suggested the potential contribution of 5hmC, the oxidized form of 5mC, to stress and psychiatric disorders. The brain shows a 10-fold enrichment of 5hmC compared to peripheral tissues, and it is associated with neuronal plasticity (Kriaucionis and Heintz, 2009; Szulwach et al., 2011). It is interesting to note that stressed animals show a sex-specific genome-wide disruption of 5hmC. Papale et al. (2016) examined the genome-wide profile of hippocampal 5hmC in female mice exposed to acute stress, and reported that 363 differentially hydroxymethylated regions are linked to known (*e.g*., *Nr3c1* and *Ntrk2*) and potentially novel genes associated with stress response and psychiatric disorders, and that stress-related hydroxymethylation is correlated with altered transcript levels. Characterization of stress-induced sex-specific 5hmC profiles identified 778 sex-specific acute stress-induced differentially hydroxymethylated regions, some of which were correlated with altered levels of transcripts that produce sex-specific isoforms in response to stress (Papale et al., 2016). Therefore, the molecular mechanisms underlying the sex-related differences in stress response may involve 5hmC modifications. Although some studies reported altered levels of 5hmC in patients with MDD and bipolar disorder (Soeiro-De-Souza et al., 2013; Tseng et al., 2014; Gross et al., 2017), further studies are needed to investigate the sex-specific role of 5hmC in psychiatric disorders.

### Roles of Non-coding RNAs in Sex-Related Differences in Depression

The domains on the X-chromosome that escape XCI include non-coding RNAs in addition to protein-coding genes (Reinius et al., 2010). Elevated expression of X chromosome miRNAs due to XCI escape has been suggested to explain sex bias of disease risk (Pinheiro et al., 2011; Gurwitz, 2019). Indeed, elevated levels of miR-221, which is located on the human X chromosome, were observed in the cerebrospinal fluid and plasma of MDD patients (Wan et al., 2015; Enatescu et al., 2016). In addition, a recent study reported elevated miR-221 expression in the cerebrospinal fluid and serum of MDD patients as well as the hippocampus of mice exposed to chronic unpredictable mild stress. Moreover, miR-221 silencing by antisense oligonucleotides was shown to improve the behavioral symptoms in a chronic stress mouse model (Lian et al., 2018). As in the immune response, the overexpression of miRNAs, such as miR-221, may explain the observed sex-related differences in MDD (Pinheiro et al., 2011). Furthermore, it has been reported that social defeat stress resulted in miR-181a-5p overexpression in the anterior bed nucleus of the stria terminalis in female but not male mice (Luo et al., 2021).

Long non-coding RNAs may also play pivotal roles in sex-specific transcriptional regulation in human depression. A recent study showed that neuronal-enriched lncRNA LINC00473 is downregulated in the PFC of depressed females but not males (Issler et al., 2020). The authors also reported that viral-mediated overexpression of LINC00473 in mouse mPFC neurons increased behavioral resilience to chronic stress, but only in females. Interestingly, LINC00473 modulates synaptic plasticity in mPFC pyramidal neurons of female mice, but not in males, and the knockdown of this lncRNA showed a stronger effect on gene expression in human female-derived neuronal cells than in those derived from males (Issler et al., 2020). LINC00473 is known to regulate CREB signaling (Chen et al., 2016; Liang et al., 2016) and CREB function is strongly associated with depression and stress resilience. Therefore, LINC00473-mediated gene expression plays a key role in molecular adaptations in the brain that contribute to depression in a sex-specific manner.

## Conclusions

Here, we briefly summarized the roles of epigenetic molecules associated with neural plasticity and behavioral regulation in animal models of depression and those that have been suggested to be involved in human MDD patients and suicide victims (Figure 3). Translational implications for bridging research in human depression and animal models will provide a better understanding of how epigenetic mechanisms contribute to the etiology and pathophysiology of depression.

Depression is a heterogeneous condition that varies widely in severity of symptoms, symptom patterns, age of onset, course trajectory, and responses to treatment, as well as showing sex-related differences. Epigenetic mechanisms, including histone modifications, DNA methylation, and non-coding RNAs, may play important roles in the pathogenesis of MDD, and these signatures may be useful as biological markers of certain subtypes of MDD. Indeed, genome-wide DNA methylation profiling analysis indicated that adult-onset and late-onset depression have distinct epigenetic signatures (Yamagata et al., 2021). In addition, there are sex-related differences in epigenetic signatures in patients with MDD as described in this article, although these findings have yet to be confirmed. Classification and identification of more homogeneous patient groups or subtypes using epigenetic marks may improve our understanding of the etiology and pathophysiology of depression with regard to patient-specific mechanisms, which may aid in the development of more biologically informed, patient-specific diagnoses and treatments (Simon and Perlis, 2010; Beijers et al., 2019).

The sex-related differences in epigenetic markers in the brain tissue are relatively subtle (Nugent et al., 2015; Shen et al., 2015). This may be because the tissues of the brain contain a multitude of cell types, and therefore sex-related differences in cell-type-specific epigenetic signatures are masked in studies relying on homogenates. There have been a few investigations of epigenetic markers in neuronal and non-neuronal nuclei obtained from brain tissues. As predicted, neuronal and non-neuronal cells show different patterns of age-related or disease-specific epigenetic changes (Cheung et al., 2010; Iwamoto et al., 2011; Shulha et al., 2013). Despite the development of conventional techniques for investigating epigenetic markers in specific cell types, further studies are necessary to characterize sex-related differences in brain epigenetics associated with stress, psychiatric symptoms, and depression.

Another limitation at present is the lack of evidence demonstrating similarities in sex-dependent mechanisms between humans and rodent models. Labonte et al. reported that human MDD and mouse chronic variable stress cohorts have an overlap in transcriptional profiles in the vmPFC and NAc. Depressed male mice and stressed mice shared 62 upregulated differentially expressed genes (DEGs) and 90 downregulated DEGs in the vmPFC and 109 upregulated DEGs and 44 downregulated DEGs in the NAc. Likewise, depressed female humans and stressed female mice shared 128 upregulated DEGs and 123 downregulated DEGs in the vmPFC and 89 upregulated DEGs and 81 downregulated DEGs in the NAc. In a gene ontology overlap analysis, several pathways in the vmPFC and NAc have been identified as interspecies conserved in males or females (Labonté et al., 2017). Despite these numerous overlaps, there are only a limited number of genes whose functions have been clarified. In addition, non-coding RNAs, including lncRNAs, could also be a key to opening the way for constructing valid animal models of human diseases and elucidating the mechanisms underlying sex-specific responses to stress in humans. Since lncRNAs are a recently discovered class of regulatory RNAs, analyzing the mechanism of action of lncRNAs remains challenging. Nevertheless, the research conducted by Issler et al. on the sex-specific role of LINC00473 in depression suggests a roadmap for future studies to explore inter-species mechanisms relating to how lncRNAs contribute to the sex differences in depression (Issler et al., 2020). A better understanding of the roles of epigenetic modifications in psychiatric disorders will be important to provide insights that will facilitate personalized therapy options as the epigenome could be modulated by genetic, age, sex, and environmental factors. Future studies must include both male and female subjects, and sex should be included as a variable to develop effective treatments for the whole population.

Depression is thought to be a heterogeneous disorder caused by deficits in multiple behavioral domains and regulated by many brain structures and systems (Russo et al., 2012). A large body of evidence has demonstrated that long-term neuroplasticity, such as structural plasticity (e.g., spine density), is associated with neuroepigenetic regulation of gene expression within the hippocampus, prefrontal cortex, and nucleus accumbens, and is essential for chronic stress susceptibility and resilience. However, very little is known about how epigenetic mechanisms in other brain regions could account for synaptic plasticity and subsequent depression risk and resilience. Recently, one study reported that the visual circuit may serve as a mechanism underlying the behavioral response to chronic stress and antidepressant actions. A recent report has shown that the activation of the retina-vLGN pathway inhibits neuronal activity in the lateral habenula (LHb), which is required for chronic stress resiliency in mice, and that light therapy ameliorated depression-like phenotypes *via* the retina-vLGN-LHb pathway (Huang et al., 2019). It was found that in rats, chronic treatment with fluoxetine reactivates visual cortex plasticity in adulthood, and this effect was accompanied, at least in part, by reduced intracortical inhibition and increased expression of BDNF in the visual cortex (Maya Vetencourt et al., 2008). In addition, the plasticity of neuronal circuitries in the visual cortex was lower in depressed patients than in control subjects and higher after chronic intake of an antidepressant (Normann et al., 2007). Interestingly, chronic treatment with fluoxetine promotes BDNF expression by promoting H3K9 acetylation in the visual cortex of rats (Maya Vetencourt et al., 2011). Although the epigenetic markers in the visual cortex of patients with MDD are unclear, these results shed new light on our current understanding of the mechanisms of brain plasticity underlying stress, depression, and antidepressant actions.

Of note, brain structure and systems may be modified in response to environmental factors in a sex-dependent way, through neurogenesis. It has been shown that female rats have greater levels of cell proliferation compared to males and non-proestrous females and this may depend on the phase of the estrous cycle (Tanapat et al., 1999). Indeed, estrogens modulate neurogenesis in females but to a lesser extent in males (Barker and Galea, 2008). BDNF signaling is one of the prime candidates for mediating neurogenesis and neural plasticity of neuronal connection (Thoenen, 1995; Castrén, 2005). As mentioned earlier, *Bdnf* expression is regulated by a variety of epigenetic modifications, including DNA methylation and histone modifications, thus suggesting that hippocampal plasticity might be influenced by neurogenesis through the epigenetic regulation of *Bdnf* expression. However, it remains unclear how plasticity and neurogenesis impact depression-related behaviors and antidepressant actions. In addition, the mystery of whether neurogenesis occurs in adult human remains to be solved (Dennis et al., 2016; Boldrini et al., 2018; Cipriani et al., 2018; Sorrells et al., 2018; Moreno-Jiménez et al., 2019). Future studies will be necessary to clarify the role of epigenetic regulation in the development and maintenance of brain networks and behaviors and their sex-related mechanisms.

## Author Contributions

All authors listed have made a substantial, direct, and intellectual contribution to the work, and have approved it for publication.

## Conflict of Interest

The authors declare that the research was conducted in the absence of any commercial or financial relationships that could be construed as a potential conflict of interest.

## References

[B1] Abe N. Uchida S. Otsuki K. Hobara T. Yamagata H. Higuchi F.. (2011). Altered sirtuin deacetylase gene expression in patients with a mood disorder. J. Psychiatr. Res. 45, 1106–1112. 10.1016/j.jpsychires.2011.01.016 21349544

[B2] Abe-Higuchi N. Uchida S. Yamagata H. Higuchi F. Hobara T. Hara K.. (2016). Hippocampal sirtuin 1 signaling mediates depression-like behavior. Biol. Psychiatry 80, 815–826. 10.1016/j.biopsych.2016.01.009 27016384

[B3] Akbarian S. Huang H. S. (2009). Epigenetic regulation in human brain-focus on histone lysine methylation. Biol. Psychiatry 65, 198–203. 10.1016/j.biopsych.2008.08.015 18814864 PMC2637452

[B4] Allen L. Dwivedi Y. (2020). MicroRNA mediators of early life stress vulnerability to depression and suicidal behavior. Mol. Psychiatry 25, 308–320. 10.1038/s41380-019-0597-8 31740756 PMC6974433

[B5] Anastas J. N. Shi Y. (2019). Histone serotonylation: can the brain have “Happy” chromatin? Mol. Cell 74, 418–420. 10.1016/j.molcel.2019.04.017 31051139 PMC6662934

[B6] Barakat T. S. Gribnau J. (2012). X chromosome inactivation in the cycle of life. Development 139, 2085–2089. 10.1242/dev.069328 22619385

[B7] Barker J. M. Galea L. A. (2008). Repeated estradiol administration alters different aspects of neurogenesis and cell death in the hippocampus of female, but not male, rats. Neuroscience 152, 888–902. 10.1016/j.neuroscience.2007.10.071 18353559

[B8] Baudry A. Mouillet-Richard S. Schneider B. Launay J. M. Kellermann O. (2010). miR-16 targets the serotonin transporter: a new facet for adaptive responses to antidepressants. Science 329, 1537–1541. 10.1126/science.1193692 20847275

[B9] Beijers L. Wardenaar K. J. Van Loo H. M. Schoevers R. A. (2019). Data-driven biological subtypes of depression: systematic review of biological approaches to depression subtyping. Mol. Psychiatry 24, 888–900. 10.1038/s41380-019-0385-5 30824865

[B10] Bicker S. Khudayberdiev S. Weiß K. Zocher K. Baumeister S. Schratt G. (2013). The DEAH-box helicase DHX36 mediates dendritic localization of the neuronal precursor-microRNA-134. Genes Dev. 27, 991–996. 10.1101/gad.211243.112 23651854 PMC3656329

[B11] Blendy J. A. (2006). The role of CREB in depression and antidepressant treatment. Biol. Psychiatry 59, 1144–1150. 10.1016/j.biopsych.2005.11.003 16457782

[B12] Boldrini M. Fulmore C. A. Tartt A. N. Simeon L. R. Pavlova I. Poposka V.. (2018). Human hippocampal neurogenesis persists throughout aging. Cell Stem Cell 22, 589.e5–599.e5. 10.1016/j.stem.2018.03.015 29625071 PMC5957089

[B13] Borrelli E. Nestler E. J. Allis C. D. Sassone-Corsi P. (2008). Decoding the epigenetic language of neuronal plasticity. Neuron 60, 961–974. 10.1016/j.neuron.2008.10.012 19109904 PMC2737473

[B14] Boulware M. I. Heisler J. D. Frick K. M. (2013). The memory-enhancing effects of hippocampal estrogen receptor activation involve metabotropic glutamate receptor signaling. J. Neurosci. 33, 15184–15194. 10.1523/JNEUROSCI.1716-13.2013 24048848 PMC6618419

[B15] Bowman R. E. Zrull M. C. Luine V. N. (2001). Chronic restraint stress enhances radial arm maze performance in female rats. Brain Res. 904, 279–289. 10.1016/s0006-8993(01)02474-x 11406126

[B16] Breslau N. Kessler R. C. Chilcoat H. D. Schultz L. R. Davis G. C. Andreski P. (1998). Trauma and posttraumatic stress disorder in the community: the 1996 Detroit Area Survey of Trauma. Arch. Gen. Psychiatry 55, 626–632. 10.1001/archpsyc.55.7.626 9672053

[B17] Carrel L. Willard H. F. (2005). X-inactivation profile reveals extensive variability in X-linked gene expression in females. Nature 434, 400–404. 10.1038/nature03479 15772666

[B18] Carrier N. Kabbaj M. (2013). Sex differences in the antidepressant-like effects of ketamine. Neuropharmacology 70, 27–34. 10.1016/j.neuropharm.2012.12.009 23337256

[B19] Carvalho-Netto E. F. Myers B. Jones K. Solomon M. B. Herman J. P. (2011). Sex differences in synaptic plasticity in stress-responsive brain regions following chronic variable stress. Physiol. Behav. 104, 242–247. 10.1016/j.physbeh.2011.01.024 21315096 PMC4486019

[B20] Casarotto P. C. Girych M. Fred S. M. Kovaleva V. Moliner R. Enkavi G.. (2021). Antidepressant drugs act by directly binding to TRKB neurotrophin receptors. Cell 184, 1299.e19–1313.e19. 10.1016/j.cell.2021.01.034 33606976 PMC7938888

[B21] Castrén E. (2005). Is mood chemistry? Nat. Rev. Neurosci. 6, 241–246. 10.1038/nrn1629 15738959

[B22] Castrén E. Hen R. (2013). Neuronal plasticity and antidepressant actions. Trends Neurosci. 36, 259–267. 10.1016/j.tins.2012.12.010 23380665 PMC3648595

[B23] Cech T. R. Steitz J. A. (2014). The noncoding RNA revolution-trashing old rules to forge new ones. Cell 157, 77–94. 10.1016/j.cell.2014.03.008 24679528

[B24] Cerniauskas I. Winterer J. De Jong J. W. Lukacsovich D. Yang H. Khan F.. (2019). Chronic stress induces activity, synaptic, and transcriptional remodeling of the lateral habenula associated with deficits in motivated behaviors. Neuron 104, 899–915 e8. 10.1016/j.neuron.2019.09.005 31672263 PMC6895430

[B25] Chahrour M. Jung S. Y. Shaw C. Zhou X. Wong S. T. Qin J.. (2008). MeCP2, a key contributor to neurological disease, activates and represses transcription. Science 320, 1224–1229. 10.1126/science.1153252 18511691 PMC2443785

[B27] Chen B. Cepko C. L. (2009). HDAC4 regulates neuronal survival in normal and diseased retinas. Science 323, 256–259. 10.1126/science.1166226 19131628 PMC3339762

[B28] Chen Z. Li J.-L. Lin S. Cao C. Gimbrone N. T. Yang R.. (2016). cAMP/CREB-regulated LINC00473 marks LKB1-inactivated lung cancer and mediates tumor growth. J. Clin. Invest. 126, 2267–2279. 10.1172/JCI85250 27140397 PMC4887185

[B26] Chen A. C. Shirayama Y. Shin K. H. Neve R. L. Duman R. S. (2001). Expression of the cAMP response element binding protein (CREB) in hippocampus produces an antidepressant effect. Biol. Psychiatry 49, 753–762. 10.1016/s0006-3223(00)01114-8 11331083

[B29] Cheung I. Shulha H. P. Jiang Y. Matevossian A. Wang J. Weng Z.. (2010). Developmental regulation and individual differences of neuronal H3K4me3 epigenomes in the prefrontal cortex. Proc. Natl. Acad. Sci. U S A 107, 8824–8829. 10.1073/pnas.1001702107 20421462 PMC2889328

[B30] Choi M. Lee S. H. Wang S. E. Ko S. Y. Song M. Choi J. S.. (2015). Ketamine produces antidepressant-like effects through phosphorylation-dependent nuclear export of histone deacetylase 5 (HDAC5) in rats. Proc. Natl. Acad. Sci. U S A 112, 15755–15760. 10.1073/pnas.1513913112 26647181 PMC4697416

[B31] Cholewa-Waclaw J. Bird A. Von Schimmelmann M. Schaefer A. Yu H. Song H.. (2016). The role of epigenetic mechanisms in the regulation of gene expression in the nervous system. J. Neurosci. 36, 11427–11434. 10.1523/JNEUROSCI.2492-16.2016 27911745 PMC5125210

[B32] Cipriani S. Ferrer I. Aronica E. Kovacs G. G. Verney C. Nardelli J.. (2018). Hippocampal radial glial subtypes and their neurogenic potential in human fetuses and healthy and Alzheimer’s disease adults. Cereb. Cortex 28, 2458–2478. 10.1093/cercor/bhy096 29722804

[B33] Coley E. J. L. Demaestri C. Ganguly P. Honeycutt J. A. Peterzell S. Rose N.. (2019). Cross-generational transmission of early life stress effects on HPA regulators and Bdnf are mediated by sex, lineage, and upbringing. Front. Behav. Neurosci. 13:101. 10.3389/fnbeh.2019.00101 31143105 PMC6521572

[B34] Conrad C. D. Grote K. A. Hobbs R. J. Ferayorni A. (2003). Sex differences in spatial and non-spatial Y-maze performance after chronic stress. Neurobiol. Learn. Mem. 79, 32–40. 10.1016/s1074-7427(02)00018-7 12482677

[B35] Conti A. C. Cryan J. F. Dalvi A. Lucki I. Blendy J. A. (2002). cAMP response element-binding protein is essential for the upregulation of brain-derived neurotrophic factor transcription, but not the behavioral or endocrine responses to antidepressant drugs. J. Neurosci. 22, 3262–3268. 10.1016/j.envpol.2021.116535 11943827 PMC6757540

[B36] Covington H. E. III. Maze I. Laplant Q. C. Vialou V. F. Ohnishi Y. N. Berton O.. (2009). Antidepressant actions of histone deacetylase inhibitors. J. Neurosci. 29, 11451–11460. 10.1523/JNEUROSCI.1758-09.2009 19759294 PMC2775805

[B37] Covington H. E. III. Maze I. Sun H. Bomze H. M. Demaio K. D. Wu E. Y.. (2011). A role for repressive histone methylation in cocaine-induced vulnerability to stress. Neuron 71, 656–670. 10.1016/j.neuron.2011.06.007 21867882 PMC3163060

[B38] Cruceanu C. Alda M. Nagy C. Freemantle E. Rouleau G. A. Turecki G. (2013). H3K4 tri-methylation in synapsin genes leads to different expression patterns in bipolar disorder and major depression. Int. J. Neuropsychopharmacol. 16, 289–299. 10.1017/S1461145712000363 22571925 PMC3564952

[B39] Cui X. Niu W. Kong L. He M. Jiang K. Chen S.. (2017). Long noncoding RNA expression in peripheral blood mononuclear cells and suicide risk in Chinese patients with major depressive disorder. Brain Behav. 7:e00711. 10.1002/brb3.711 28638716 PMC5474714

[B40] Dandi E. Kalamari A. Touloumi O. Lagoudaki R. Nousiopoulou E. Simeonidou C.. (2018). Beneficial effects of environmental enrichment on behavior, stress reactivity and synaptophysin/BDNF expression in hippocampus following early life stress. Int. J. Dev. Neurosci. 67, 19–32. 10.1016/j.ijdevneu.2018.03.003 29545098

[B41] Dennis C. V. Suh L. S. Rodriguez M. L. Kril J. J. Sutherland G. T. (2016). Human adult neurogenesis across the ages: An immunohistochemical study. Neuropathol. Appl. Neurobiol. 42, 621–638. 10.1111/nan.12337 27424496 PMC5125837

[B42] Dias C. Feng J. Sun H. Shao N. Y. Mazei-Robison M. S. Damez-Werno D.. (2014). β-catenin mediates stress resilience through Dicer1/microRNA regulation. Nature 516, 51–55. 10.1038/nature13976 25383518 PMC4257892

[B43] Drevets W. C. (2001). Neuroimaging and neuropathological studies of depression: implications for the cognitive-emotional features of mood disorders. Curr. Opin. Neurobiol. 11, 240–249. 10.1016/s0959-4388(00)00203-8 11301246

[B44] Dworkin A. M. Huang T. H.-M. Toland A. E. (2009). Epigenetic alterations in the breast: implications for breast cancer detection, prognosis and treatment. Semin. Cancer Biol. 19, 165–171. 10.1016/j.semcancer.2009.02.007 19429480 PMC2734184

[B45] Enatescu V. R. Papava I. Enatescu I. Antonescu M. Anghel A. Seclaman E.. (2016). Circulating plasma micro RNAs in patients with major depressive disorder treated with antidepressants: a pilot study. Psychiatry Investig. 13, 549–557. 10.4306/pi.2016.13.5.549 27757134 PMC5067350

[B46] Fagundes C. P. Glaser R. Kiecolt-Glaser J. K. (2013). Stressful early life experiences and immune dysregulation across the lifespan. Brain Behav. Immun. 27, 8–12. 10.1016/j.bbi.2012.06.014 22771426 PMC3518756

[B47] Fan C. Zhu X. Song Q. Wang P. Liu Z. Yu S. Y. (2018). MiR-134 modulates chronic stress-induced structural plasticity and depression-like behaviors *via* downregulation of Limk1/cofilin signaling in rats. Neuropharmacology 131, 364–376. 10.1016/j.neuropharm.2018.01.009 29329879

[B48] Fang Y. Qiu Q. Zhang S. Sun L. Li G. Xiao S.. (2018). Changes in miRNA-132 and miR-124 levels in non-treated and citalopram-treated patients with depression. J. Affect. Disord. 227, 745–751. 10.1016/j.jad.2017.11.090 29689690

[B49] Farrelly L. A. Thompson R. E. Zhao S. Lepack A. E. Lyu Y. Bhanu N. V.. (2019). Histone serotonylation is a permissive modification that enhances TFIID binding to H3K4me3. Nature 567, 535–539. 10.1038/s41586-019-1024-7 30867594 PMC6557285

[B50] Ferguson A. V. Latchford K. J. Samson W. K. (2008). The paraventricular nucleus of the hypothalamus - a potential target for integrative treatment of autonomic dysfunction. Expert Opin. Ther. Targets 12, 717–727. 10.1517/14728222.12.6.717 18479218 PMC2682920

[B51] Fernández-Guasti A. Fiedler J. L. Herrera L. Handa R. J. (2012). Sex, stress, and mood disorders: at the intersection of adrenal and gonadal hormones. Horm. Metab. Res. 44, 607–618. 10.1055/s-0032-1312592 22581646 PMC3584173

[B52] Figueiredo H. F. Dolgas C. M. Herman J. P. (2002). Stress activation of cortex and hippocampus is modulated by sex and stage of estrus. Endocrinology 143, 2534–2540. 10.1210/endo.143.7.8888 12072385

[B53] Flavell S. W. Greenberg M. E. (2008). Signaling mechanisms linking neuronal activity to gene expression and plasticity of the nervous system. Annu. Rev. Neurosci. 31, 563–590. 10.1146/annurev.neuro.31.060407.125631 18558867 PMC2728073

[B54] Franceschelli A. Sens J. Herchick S. Thelen C. Pitychoutis P. M. (2015). Sex differences in the rapid and the sustained antidepressant-like effects of ketamine in stress-naive and “depressed” mice exposed to chronic mild stress. Neuroscience 290, 49–60. 10.1016/j.neuroscience.2015.01.008 25595985

[B55] Fuchikami M. Morinobu S. Segawa M. Okamoto Y. Yamawaki S. Ozaki N.. (2011). DNA methylation profiles of the brain-derived neurotrophic factor (BDNF) gene as a potent diagnostic biomarker in major depression. PLoS One 6:e23881. 10.1371/journal.pone.0023881 21912609 PMC3166055

[B56] Furmark T. Tillfors M. Marteinsdottir I. Fischer H. Pissiota A. Langstrom B.. (2002). Common changes in cerebral blood flow in patients with social phobia treated with citalopram or cognitive-behavioral therapy. Arch. Gen. Psychiatry 59, 425–433. 10.1001/archpsyc.59.5.425 11982446

[B57] Galea L. A. McEwen B. S. Tanapat P. Deak T. Spencer R. L. Dhabhar F. S. (1997). Sex differences in dendritic atrophy of CA3 pyramidal neurons in response to chronic restraint stress. Neuroscience 81, 689–697. 10.1016/s0306-4522(97)00233-9 9316021

[B58] Gao J. Wang W.-Y. Mao Y.-W. Graff J. Guan J.-S. Pan L.. (2010). A novel pathway regulates memory and plasticity *via* SIRT1 and miR-134. Nature 466, 1105–1109. 10.1038/nature09271 20622856 PMC2928875

[B59] Golden S. A. Christoffel D. J. Heshmati M. Hodes G. E. Magida J. Davis K.. (2013). Epigenetic regulation of RAC1 induces synaptic remodeling in stress disorders and depression. Nat. Med. 19, 337–344. 10.1038/nm.3090 23416703 PMC3594624

[B60] Gould E. Woolley C. S. Frankfurt M. McEwen B. S. (1990). Gonadal steroids regulate dendritic spine density in hippocampal pyramidal cells in adulthood. J. Neurosci. 10, 1286–1291. 10.1523/JNEUROSCI.10-04-01286.1990 2329377 PMC6570209

[B61] Gräff J. Rei D. Guan J.-S. Wang W.-Y. Seo J. Hennig K. M.. (2012). An epigenetic blockade of cognitive functions in the neurodegenerating brain. Nature 483, 222–226. 10.1038/nature10849 22388814 PMC3498952

[B62] Greer P. L. Greenberg M. E. (2008). From synapse to nucleus: calcium-dependent gene transcription in the control of synapse development and function. Neuron 59, 846–860. 10.1016/j.neuron.2008.09.002 18817726

[B63] Gross J. A. Pacis A. Chen G. G. Drupals M. Lutz P.-E. Barreiro L. B.. (2017). Gene-body 5-hydroxymethylation is associated with gene expression changes in the prefrontal cortex of depressed individuals. Transl. Psychiatry 7:e1119. 10.1038/tp.2017.93 28485726 PMC5534961

[B64] Guan J.-S. Haggarty S. J. Giacometti E. Dannenberg J.-H. Joseph N. Gao J.. (2009). HDAC2 negatively regulates memory formation and synaptic plasticity. Nature 459, 55–60. 10.1038/nature07925 19424149 PMC3498958

[B65] Gurwitz D. (2019). Genomics and the future of psychopharmacology: MicroRNAs offer novel therapeutics. Dialogues Clin. Neurosci. 21, 131–148. 10.31887/DCNS.2019.21.2/dgurwitz 31636487 PMC6787538

[B66] Hammen C. (2005). Stress and depression. Annu. Rev. Clin. Psychol. 1, 293–319. 10.1146/annurev.clinpsy.1.102803.143938 17716090

[B67] Harrison P. J. (2002). The neuropathology of primary mood disorder. Brain 125, 1428–1449. 10.1093/brain/awf149 12076995

[B68] He C. Bai Y. Wang Z. Fan D. Wang Q. Liu X.. (2021). Identification of microRNA-9 linking the effects of childhood maltreatment on depression using amygdala connectivity. NeuroImage 224:117428. 10.1016/j.neuroimage.2020.117428 33038536

[B69] He S. Liu X. Jiang K. Peng D. Hong W. Fang Y.. (2016). Alterations of microRNA-124 expression in peripheral blood mononuclear cells in pre- and post-treatment patients with major depressive disorder. J. Psychiatr. Res. 78, 65–71. 10.1016/j.jpsychires.2016.03.015 27078210

[B70] Heck A. L. Handa R. J. (2019). Sex differences in the hypothalamic-pituitary-adrenal axis’ response to stress: an important role for gonadal hormones. Neuropsychopharmacology 44, 45–58. 10.1038/s41386-018-0167-9 30111811 PMC6235871

[B71] Heim C. Nemeroff C. B. (2001). The role of childhood trauma in the neurobiology of mood and anxiety disorders: preclinical and clinical studies. Biol. Psychiatry 49, 1023–1039. 10.1016/s0006-3223(01)01157-x 11430844

[B72] Higuchi F. Uchida S. Yamagata H. Abe-Higuchi N. Hobara T. Hara K.. (2016). Hippocampal MicroRNA-124 enhances chronic stress resilience in mice. J. Neurosci. 36, 7253–7267. 10.1523/JNEUROSCI.0319-16.2016 27383599 PMC6705534

[B73] Higuchi F. Uchida S. Yamagata H. Otsuki K. Hobara T. Abe N.. (2011). State-dependent changes in the expression of DNA methyltransferases in mood disorder patients. J. Psychiatr. Res. 45, 1295–1300. 10.1016/j.jpsychires.2011.04.008 21592522

[B74] Hill J. Pickles A. Wright N. Quinn J. P. Murgatroyd C. Sharp H. (2019). Mismatched prenatal and postnatal maternal depressive symptoms and child behaviours: a sex-dependent role for NR3C1 DNA methylation in the wirral child health and development study. Cells 8:943. 10.3390/cells8090943 31438539 PMC6770436

[B75] Hirata T. Otsuka I. Okazaki S. Mouri K. Horai T. Boku S.. (2019). Major depressive disorder-associated SIRT1 locus affects the risk for suicide in women after middle age. Psychiatry Res. 278, 141–145. 10.1016/j.psychres.2019.06.002 31176830

[B76] Hobara T. Uchida S. Otsuki K. Matsubara T. Funato H. Matsuo K.. (2010). Altered gene expression of histone deacetylases in mood disorder patients. J. Psychiatr. Res. 44, 263–270. 10.1016/j.jpsychires.2009.08.015 19767015

[B77] Hodes G. E. Pfau M. L. Purushothaman I. Ahn H. F. Golden S. A. Christoffel D. J.. (2015). Sex differences in nucleus accumbens transcriptome profiles associated with susceptibility versus resilience to subchronic variable stress. J. Neurosci. 35, 16362–16376. 10.1523/JNEUROSCI.1392-15.2015 26674863 PMC4679819

[B78] Howard D. M. Adams M. J. Clarke T. K. Hafferty J. D. Gibson J. Shirali M.. (2019). Genome-wide meta-analysis of depression identifies 102 independent variants and highlights the importance of the prefrontal brain regions. Nat. Neurosci. 22, 343–352. 10.1038/s41593-018-0326-7 30718901 PMC6522363

[B79] Hu Z. Yu D. Gu Q.-H. Yang Y. Tu K. Zhu J.. (2014). miR-191 and miR-135 are required for long-lasting spine remodelling associated with synaptic long-term depression. Nat. Commun. 5:3263. 10.1038/ncomms4263 24535612 PMC3951436

[B80] Huang L. Xi Y. Peng Y. Yang Y. Huang X. Fu Y.. (2019). A visual circuit related to habenula underlies the antidepressive effects of light therapy. Neuron 102, 128.e8–142.e8. 10.1016/j.neuron.2019.01.037 30795900

[B81] Hyer M. M. Phillips L. L. Neigh G. N. (2018). Sex differences in synaptic plasticity: hormones and beyond. Front. Mol. Neurosci. 11:266. 10.3389/fnmol.2018.00266 30108482 PMC6079238

[B82] Iga J.-I. Ueno S.-I. Yamauchi K. Numata S. Kinouchi S. Tayoshi-Shibuya S.. (2007). Altered HDAC5 and CREB mRNA expressions in the peripheral leukocytes of major depression. Prog. Neuropsychopharmacol. Biol. Psychiatry 31, 628–632. 10.1016/j.pnpbp.2006.12.014 17258370

[B83] Inoue A. Jiang L. Lu F. Suzuki T. Zhang Y. (2017). Maternal H3K27me3 controls DNA methylation-independent imprinting. Nature 547, 419–424. 10.1038/nature23262 28723896 PMC9674007

[B84] Issler O. Haramati S. Paul E. D. Maeno H. Navon I. Zwang R.. (2014). MicroRNA 135 is essential for chronic stress resiliency, antidepressant efficacy, and intact serotonergic activity. Neuron 83, 344–360. 10.1016/j.neuron.2014.05.042 24952960

[B85] Issler O. Van Der Zee Y. Y. Ramakrishnan A. Wang J. Tan C. Loh Y.-E.. (2020). Sex-specific role for the long non-coding RNA LINC00473 in depression. Neuron 106, 912.e5–926.e5. 10.1016/j.neuron.2020.03.023 32304628 PMC7305959

[B86] Iwamoto K. Bundo M. Ueda J. Oldham M. C. Ukai W. Hashimoto E.. (2011). Neurons show distinctive DNA methylation profile and higher interindividual variations compared with non-neurons. Genome Res. 21, 688–696. 10.1101/gr.112755.110 21467265 PMC3083085

[B87] Jaenisch R. Bird A. (2003). Epigenetic regulation of gene expression: how the genome integrates intrinsic and environmental signals. Nat. Genet. 33, 245–254. 10.1038/ng1089 12610534

[B88] Ji B. Higa K. K. Kelsoe J. R. Zhou X. (2015). Over-expression of XIST, the master gene for X chromosome inactivation, in females with major affective disorders. EBioMedicine 2, 909–918. 10.1016/j.ebiom.2015.06.012 26425698 PMC4563114

[B89] Johnston C. M. Lovell F. L. Leongamornlert D. A. Stranger B. E. Dermitzakis E. T. Ross M. T. (2008). Large-scale population study of human cell lines indicates that dosage compensation is virtually complete. PLoS Genet. 4:e9. 10.1371/journal.pgen.0040009 18208332 PMC2213701

[B90] Juruena M. F. Gadelrab R. Cleare A. J. Young A. H. (2020). Epigenetics: a missing link between early life stress and depression. Prog. Neuropsychopharmacol. Biol. Psychiatry 109:110231. 10.1016/j.pnpbp.2020.110231 33383101

[B91] Kang H. J. Voleti B. Hajszan T. Rajkowska G. Stockmeier C. A. Licznerski P.. (2012). Decreased expression of synapse-related genes and loss of synapses in major depressive disorder. Nat. Med. 18, 1413–1417. 10.1038/nm.2886 22885997 PMC3491115

[B92] Karpova N. N. Pickenhagen A. Lindholm J. Tiraboschi E. Kulesskaya N. Agustsdottir A.. (2011). Fear erasure in mice requires synergy between antidepressant drugs and extinction training. Science 334, 1731–1734. 10.1126/science.1214592 22194582 PMC3929964

[B93] Kelly M. J. Levin E. R. (2001). Rapid actions of plasma membrane estrogen receptors. Trends Endocrinol. Metab. 12, 152–156. 10.1016/s1043-2760(01)00377-0 11295570

[B94] Kessler R. C. Chiu W. T. Demler O. Merikangas K. R. Walters E. E. (2005). Prevalence, severity, and comorbidity of 12-month DSM-IV disorders in the National Comorbidity Survey Replication. Arch. Gen. Psychiatry 62, 617–627. 10.1001/archpsyc.62.6.617 15939839 PMC2847357

[B95] Kessler R. C. McGonagle K. A. Swartz M. Blazer D. G. Nelson C. B. (1993). Sex and depression in the National Comorbidity Survey. I: lifetime prevalence, chronicity and recurrence. J. Affect. Disord. 29, 85–96. 10.1016/0165-0327(93)90026-g 8300981

[B96] Khan A. Brodhead A. E. Schwartz K. A. Kolts R. L. Brown W. A. (2005). Sex differences in antidepressant response in recent antidepressant clinical trials. J. Clin. Psychopharmacol. 25, 318–324. 10.1097/01.jcp.0000168879.03169.ce 16012273

[B97] Kim D. R. Bale T. L. Epperson C. N. (2015). Prenatal programming of mental illness: current understanding of relationship and mechanisms. Curr. Psychiatry Rep. 17:5. 10.1007/s11920-014-0546-9 25617041 PMC4458064

[B98] Kim M. S. Akhtar M. W. Adachi M. Mahgoub M. Bassel-Duby R. Kavalali E. T.. (2012). An essential role for histone deacetylase 4 in synaptic plasticity and memory formation. J. Neurosci. 32, 10879–10886. 10.1523/JNEUROSCI.2089-12.2012 22875922 PMC3480333

[B99] Kishi T. Yoshimura R. Kitajima T. Okochi T. Okumura T. Tsunoka T.. (2010). SIRT1 gene is associated with major depressive disorder in the Japanese population. J. Affect. Disord. 126, 167–173. 10.1016/j.jad.2010.04.003 20451257

[B100] Kobayashi K. Ikeda Y. Sakai A. Yamasaki N. Haneda E. Miyakawa T.. (2010). Reversal of hippocampal neuronal maturation by serotonergic antidepressants. Proc. Natl. Acad. Sci. U S A 107, 8434–8439. 10.1073/pnas.0912690107 20404165 PMC2889553

[B101] Kornstein S. G. Schatzberg A. F. Thase M. E. Yonkers K. A. McCullough J. P. Keitner G. I.. (2000a). Gender differences in treatment response to sertraline versus imipramine in chronic depression. Am. J. Psychiatry 157, 1445–1452. 10.1176/appi.ajp.157.9.1445 10964861

[B102] Kornstein S. G. Schatzberg A. F. Thase M. E. Yonkers K. A. Mccullough J. P. Keitner G. I.. (2000b). Gender differences in chronic major and double depression. J. Affect. Disord. 60, 1–11. 10.1016/s0165-0327(99)00158-5 10940442

[B103] Kosik K. S. (2006). The neuronal microRNA system. Nat. Rev. Neurosci. 7, 911–920. 10.1038/nrn2037 17115073

[B104] Kouzarides T. (2007). Chromatin modifications and their function. Cell 128, 693–705. 10.1016/j.cell.2007.02.005 17320507

[B105] Kriaucionis S. Heintz N. (2009). The nuclear DNA base 5-hydroxymethylcytosine is present in Purkinje neurons and the brain. Science 324, 929–930. 10.1126/science.1169786 19372393 PMC3263819

[B106] Krishnan V. Han M.-H. Graham D. L. Berton O. Renthal W. Russo S. J.. (2007). Molecular adaptations underlying susceptibility and resistance to social defeat in brain reward regions. Cell 131, 391–404. 10.1016/j.cell.2007.09.018 17956738

[B107] Kurian J. R. Olesen K. M. Auger A. P. (2010). Sex differences in epigenetic regulation of the estrogen receptor-α promoter within the developing preoptic area. Endocrinology 151, 2297–2305. 10.1210/en.2009-0649 20237133 PMC2869250

[B108] Labonté B. Engmann O. Purushothaman I. Menard C. Wang J. Tan C.. (2017). Sex-specific transcriptional signatures in human depression. Nat. Med. 23, 1102–1111. 10.1038/nm.4386 28825715 PMC5734943

[B109] LaPlant Q. Vialou V. Covington H. E. III. Dumitriu D. Feng J. Warren B. L.. (2010). Dnmt3a regulates emotional behavior and spine plasticity in the nucleus accumbens. Nat. Neurosci. 13, 1137–1143. 10.1038/nn.2619 20729844 PMC2928863

[B110] Lau P. Verrier J. D. Nielsen J. A. Johnson K. R. Notterpek L. Hudson L. D. (2008). Identification of dynamically regulated microRNA and mRNA networks in developing oligodendrocytes. J. Neurosci. 28, 11720–11730. 10.1523/JNEUROSCI.1932-08.2008 18987208 PMC2646797

[B111] Ledford H. (2015). First robust genetic links to depression emerge. Nature 523, 268–269. 10.1038/523268a 26178945

[B112] Leistedt S. J. Linkowski P. (2013). Brain, networks, depression, and more. Eur. Neuropsychopharmacol. 23, 55–62. 10.1016/j.euroneuro.2012.10.011 23154052

[B113] Lepack A. E. Werner C. T. Stewart A. F. Fulton S. L. Zhong P. Farrelly L. A.. (2020). Dopaminylation of histone H3 in ventral tegmental area regulates cocaine seeking. Science 368, 197–201. 10.1126/science.aaw8806 32273471 PMC7228137

[B114] Leranth C. Petnehazy O. MacLusky N. J. (2003). Gonadal hormones affect spine synaptic density in the CA1 hippocampal subfield of male rats. J. Neurosci. 23, 1588–1592. 10.1523/JNEUROSCI.23-05-01588.2003 12629162 PMC6741990

[B115] Li C. Brake W. G. Romeo R. D. Dunlop J. C. Gordon M. Buzescu R.. (2004). Estrogen alters hippocampal dendritic spine shape and enhances synaptic protein immunoreactivity and spatial memory in female mice. Proc. Natl. Acad. Sci. U S A 101, 2185–2190. 10.1073/pnas.0307313101 14766964 PMC357073

[B116] Lian N. Niu Q. Lei Y. Li X. Li Y. Song X. (2018). MiR-221 is involved in depression by regulating Wnt2/CREB/BDNF axis in hippocampal neurons. Cell Cycle 17, 2745–2755. 10.1080/15384101.2018.1556060 30589396 PMC6343714

[B117] Liang X.-H. Deng W.-B. Liu Y.-F. Liang Y.-X. Fan Z.-M. Gu X.-W.. (2016). Non-coding RNA LINC00473 mediates decidualization of human endometrial stromal cells in response to cAMP signaling. Sci. Rep. 6:22744. 10.1038/srep22744 26947914 PMC4780002

[B118] Lister R. Mukamel E. A. Nery J. R. Urich M. Puddifoot C. A. Johnson N. D.. (2013). Global epigenomic reconfiguration during mammalian brain development. Science 341:1237905. 10.1126/science.1237905 23828890 PMC3785061

[B119] Liu J. Hu P. Qi X. R. Meng F. T. Kalsbeek A. Zhou J. N. (2011). Acute restraint stress increases intrahypothalamic oestradiol concentrations in conjunction with increased hypothalamic oestrogen receptor β and aromatase mRNA expression in female rats. J. Neuroendocrinol. 23, 435–443. 10.1111/j.1365-2826.2011.02123.x 21392135

[B120] Lonze B. E. Ginty D. D. (2002). Function and regulation of CREB family transcription factors in the nervous system. Neuron 35, 605–623. 10.1016/s0896-6273(02)00828-0 12194863

[B121] Lopez J. P. Fiori L. M. Cruceanu C. Lin R. Labonte B. Cates H. M.. (2017). MicroRNAs 146a/b-5 and 425–3p and 24-3p are markers of antidepressant response and regulate MAPK/Wnt-system genes. Nat. Commun. 8:15497. 10.1038/ncomms15497 28530238 PMC5477510

[B122] Lopez J. P. Lim R. Cruceanu C. Crapper L. Fasano C. Labonte B.. (2014). miR-1202 is a primate-specific and brain-enriched microRNA involved in major depression and antidepressant treatment. Nat. Med. 20, 764–768. 10.1038/nm.3582 24908571 PMC4087015

[B123] Lugli G. Larson J. Martone M. E. Jones Y. Smalheiser N. R. (2005). Dicer and eIF2c are enriched at postsynaptic densities in adult mouse brain and are modified by neuronal activity in a calpain-dependent manner. J. Neurochem. 94, 896–905. 10.1111/j.1471-4159.2005.03224.x 16092937

[B124] Lugli G. Torvik V. I. Larson J. Smalheiser N. R. (2008). Expression of microRNAs and their precursors in synaptic fractions of adult mouse forebrain. J. Neurochem. 106, 650–661. 10.1111/j.1471-4159.2008.05413.x 18410515 PMC3711666

[B125] Luine V. (2002). Sex differences in chronic stress effects on memory in rats. Stress 5, 205–216. 10.1080/1025389021000010549 12186683

[B126] Lund T. D. Hinds L. R. Handa R. J. (2006). The androgen 5α-dihydrotestosterone and its metabolite 5α-androstan-3β, 17β-diol inhibit the hypothalamo-pituitary-adrenal response to stress by acting through estrogen receptor β-expressing neurons in the hypothalamus. J. Neurosci. 26, 1448–1456. 10.1523/JNEUROSCI.3777-05.2006 16452668 PMC6675494

[B127] Luo P. X. Manning C. E. Fass J. N. Williams A. V. Hao R. Campi K. L.. (2021). Sex-specific effects of social defeat stress on miRNA expression in the anterior BNST. Behav. Brain Res. 401:113084. 10.1016/j.bbr.2020.113084 33358922 PMC7864284

[B128] Luo X.-J. Zhang C. (2016). Down-regulation of SIRT1 gene expression in major depressive disorder. Am. J. Psychiatry 173:1046. 10.1176/appi.ajp.2016.16040394 27690561

[B129] Lutz P. E. Tanti A. Gasecka A. Barnett-Burns S. Kim J. J. Zhou Y.. (2017). Association of a history of child abuse with impaired myelination in the anterior cingulate cortex: convergent epigenetic, transcriptional, and morphological evidence. Am. J. Psychiatry 174, 1185–1194. 10.1176/appi.ajp.2017.16111286 28750583

[B130] MacQueen G. M. Memedovich K. A. (2017). Cognitive dysfunction in major depression and bipolar disorder: assessment and treatment options. Psychiatry Clin. Neurosci. 71, 18–27. 10.1111/pcn.12463 27685435

[B131] Maddox S. A. Kilaru V. Shin J. Jovanovic T. Almli L. M. Dias B. G.. (2018). Estrogen-dependent association of HDAC4 with fear in female mice and women with PTSD. Mol. Psychiatry 23, 658–665. 10.1038/mp.2016.250 28093566 PMC5513798

[B132] Malhi G. S. Mann J. J. (2018). Depression. Lancet 392, 2299–2312. 10.1016/S0140-6736(18)31948-2 30396512

[B133] Manners M. T. Brynildsen J. K. Schechter M. Liu X. Eacret D. Blendy J. A. (2019). CREB deletion increases resilience to stress and downregulates inflammatory gene expression in the hippocampus. Brain Behav. Immun. 81, 388–398. 10.1016/j.bbi.2019.06.035 31255680 PMC6754757

[B134] Marcus S. M. Young E. A. Kerber K. B. Kornstein S. Farabaugh A. H. Mitchell J.. (2005). Gender differences in depression: findings from the STAR*D study. J. Affect. Disord. 87, 141–150. 10.1016/j.jad.2004.09.008 15982748

[B135] Marrocco J. McEwen B. S. (2016). Sex in the brain: hormones and sex differences. Dialogues Clin. Neurosci. 18, 373–383. 10.31887/DCNS.2016.18.4/jmarrocco 28179809 PMC5286723

[B136] Matsuda K. I. Mori H. Nugent B. M. Pfaff D. W. Mccarthy M. M. Kawata M. (2011). Histone deacetylation during brain development is essential for permanent masculinization of sexual behavior. Endocrinology 152, 2760–2767. 10.1210/en.2011-0193 21586557 PMC3115610

[B137] Maya Vetencourt J. F. Sale A. Viegi A. Baroncelli L. De Pasquale R. O’leary O. F.. (2008). The antidepressant fluoxetine restores plasticity in the adult visual cortex. Science 320, 385–388. 10.1126/science.1150516 18420937

[B138] Maya Vetencourt J. F. Tiraboschi E. Spolidoro M. Castren E. Maffei L. (2011). Serotonin triggers a transient epigenetic mechanism that reinstates adult visual cortex plasticity in rats. Eur. J. Neurosci. 33, 49–57. 10.1111/j.1460-9568.2010.07488.x 21156002

[B139] Mayberg H. S. Liotti M. Brannan S. K. McGinnis S. Mahurin R. K. Jerabek P. A.. (1999). Reciprocal limbic-cortical function and negative mood: converging PET findings in depression and normal sadness. Am. J. Psychiatry 156, 675–682. 10.1176/ajp.156.5.675 10327898

[B140] Maze I. Noh K.-M. Allis C. D. (2013). Histone regulation in the CNS: basic principles of epigenetic plasticity. Neuropsychopharmacology 38, 3–22. 10.1038/npp.2012.124 22828751 PMC3521967

[B141] MccLung C. A. Nestler E. J. (2008). Neuroplasticity mediated by altered gene expression. Neuropsychopharmacology 33, 3–17. 10.1038/sj.npp.1301544 17728700

[B142] McEwen B. S. (1999). Stress and hippocampal plasticity. Annu. Rev. Neurosci. 22, 105–122. 10.1146/annurev.neuro.22.1.105 10202533

[B143] McEwen B. S. Nasca C. Gray J. D. (2016). Stress effects on neuronal structure: hippocampus, amygdala, and prefrontal cortex. Neuropsychopharmacology 41, 3–23. 10.1038/npp.2015.171 26076834 PMC4677120

[B144] McGowan P. O. Sasaki A. D’Alessio A. C. Dymov S. Labonté B. Szyf M.. (2009). Epigenetic regulation of the glucocorticoid receptor in human brain associates with childhood abuse. Nat. Neurosci. 12, 342–348. 10.1038/nn.2270 19234457 PMC2944040

[B145] Miller W. J. Suzuki S. Miller L. K. Handa R. Uht R. M. (2004). Estrogen receptor (ER)β isoforms rather than ERα regulate corticotropin-releasing hormone promoter activity through an alternate pathway. J. Neurosci. 24, 10628–10635. 10.1523/JNEUROSCI.5540-03.2004 15564578 PMC6730133

[B146] Modarresi F. Faghihi M. A. Lopez-Toledano M. A. Fatemi R. P. Magistri M. Brothers S. P.. (2012). Inhibition of natural antisense transcripts *in vivo* results in gene-specific transcriptional upregulation. Nat. Biotechnol. 30, 453–459. 10.1038/nbt.2158 22446693 PMC4144683

[B147] Moda-Sava R. N. Murdock M. H. Parekh P. K. Fetcho R. N. Huang B. S. Huynh T. N.. (2019). Sustained rescue of prefrontal circuit dysfunction by antidepressant-induced spine formation. Science 364:eaat8078. 10.1126/science.aat8078 30975859 PMC6785189

[B148] Moreno-Jiménez E. P. Flor-García M. Terreros-Roncal J. Rábano A. Cafini F. Pallas-Bazarra N.. (2019). Adult hippocampal neurogenesis is abundant in neurologically healthy subjects and drops sharply in patients with Alzheimer’s disease. Nat. Med. 25, 554–560. 10.1038/s41591-019-0375-9 30911133

[B149] Muda M. Theodosiou A. Rodrigues N. Boschert U. Camps M. Gillieron C.. (1996). The dual specificity phosphatases M3/6 and MKP-3 are highly selective for inactivation of distinct mitogen-activated protein kinases. J. Biol. Chem. 271, 27205–27208. 10.1074/jbc.271.44.27205 8910287

[B150] Nagy C. Maitra M. Tanti A. Suderman M. Theroux J. F. Davoli M. A.. (2020). Single-nucleus transcriptomics of the prefrontal cortex in major depressive disorder implicates oligodendrocyte precursor cells and excitatory neurons. Nat. Neurosci. 23, 771–781. 10.1038/s41593-020-0621-y 32341540

[B151] Nagy C. Suderman M. Yang J. Szyf M. Mechawar N. Ernst C.. (2015). Astrocytic abnormalities and global DNA methylation patterns in depression and suicide. Mol. Psychiatry 20, 320–328. 10.1038/mp.2014.21 24662927 PMC5293540

[B152] Nagy C. Torres-Platas S. G. Mechawar N. Turecki G. (2017). Repression of astrocytic connexins in cortical and subcortical brain regions and prefrontal enrichment of H3K9me3 in depression and suicide. Int. J. Neuropsychopharmacol. 20, 50–57. 10.1093/ijnp/pyw071 27516431 PMC5737582

[B153] Nestler E. J. (2014). Epigenetic mechanisms of depression. JAMA Psychiatry 71, 454–456. 10.1001/jamapsychiatry.2013.4291 24499927 PMC4057796

[B154] Nestler E. J. Barrot M. DiLeone R. J. Eisch A. J. Gold S. J. Monteggia L. M. (2002). Neurobiology of depression. Neuron 34, 13–25. 10.1016/s0896-6273(02)00653-0 11931738

[B155] Nestler E. J. Peña C. J. Kundakovic M. Mitchell A. Akbarian S. (2016). Epigenetic basis of mental illness. Neuroscientist 22, 447–463. 10.1177/1073858415608147 26450593 PMC4826318

[B156] Network and Pathway Analysis Subgroup of Psychiatric Genomics Consortium. (2015). Psychiatric genome-wide association study analyses implicate neuronal, immune and histone pathways. Nat. Neurosci. 18, 199–209. 10.1038/nn.3922 25599223 PMC4378867

[B157] Ng S.-Y. Lin L. Soh B. S. Stanton L. W. (2013). Long noncoding RNAs in development and disease of the central nervous system. Trends Genet. 29, 461–468. 10.1016/j.tig.2013.03.002 23562612

[B158] Nibuya M. Nestler E. J. Duman R. S. (1996). Chronic antidepressant administration increases the expression of cAMP response element binding protein (CREB) in rat hippocampus. J. Neurosci. 16, 2365–2372. 10.1523/JNEUROSCI.16-07-02365.1996 8601816 PMC6578518

[B159] Nie X. Kitaoka S. Tanaka K. Segi-Nishida E. Imoto Y. Ogawa A.. (2018). The innate immune receptors TLR2/4 mediate repeated social defeat stress-induced social avoidance through prefrontal microglial activation. Neuron 99, 464.e7–479.e7. 10.1016/j.neuron.2018.06.035 30033154

[B160] Nishiyama A. Yamaguchi L. Sharif J. Johmura Y. Kawamura T. Nakanishi K.. (2013). Uhrf1-dependent H3K23 ubiquitylation couples maintenance DNA methylation and replication. Nature 502, 249–253. 10.1038/nature12488 24013172

[B161] Normann C. Schmitz D. Fürmaier A. Döing C. Bach M. (2007). Long-term plasticity of visually evoked potentials in humans is altered in major depression. Biol. Psychiatry 62, 373–380. 10.1016/j.biopsych.2006.10.006 17240361

[B162] Nugent B. M. Wright C. L. Shetty A. C. Hodes G. E. Lenz K. M. Mahurkar A.. (2015). Brain feminization requires active repression of masculinization *via* DNA methylation. Nat. Neurosci. 18, 690–697. 10.1038/nn.3988 25821913 PMC4519828

[B163] Oberlander J. G. Woolley C. S. (2016). 17β-estradiol acutely potentiates glutamatergic synaptic transmission in the hippocampus through distinct mechanisms in males and females. J. Neurosci. 36, 2677–2690. 10.1523/JNEUROSCI.4437-15.2016 26937008 PMC4879212

[B164] O’Connor R. M. Grenham S. Dinan T. G. Cryan J. F. (2013). microRNAs as novel antidepressant targets: converging effects of ketamine and electroconvulsive shock therapy in the rat hippocampus. Int. J. Neuropsychopharmacol. 16, 1885–1892. 10.1017/S1461145713000448 23684180

[B165] Okada S. Morinobu S. Fuchikami M. Segawa M. Yokomaku K. Kataoka T.. (2014). The potential of SLC6A4 gene methylation analysis for the diagnosis and treatment of major depression. J. Psychiatr. Res. 53, 47–53. 10.1016/j.jpsychires.2014.02.002 24657235

[B166] Ormel J. Hartman C. A. Snieder H. (2019). The genetics of depression: successful genome-wide association studies introduce new challenges. Transl. Psychiatry 9:114. 10.1038/s41398-019-0450-5 30877272 PMC6420566

[B167] Ortiz J. B. Taylor S. B. Hoffman A. N. Campbell A. N. Lucas L. R. Conrad C. D. (2015). Sex-specific impairment and recovery of spatial learning following the end of chronic unpredictable restraint stress: potential relevance of limbic GAD. Behav. Brain Res. 282, 176–184. 10.1016/j.bbr.2014.12.051 25591480 PMC4324367

[B168] Otsuki K. Uchida S. Watanuki T. Wakabayashi Y. Fujimoto M. Matsubara T.. (2008). Altered expression of neurotrophic factors in patients with major depression. J. Psychiatr. Res. 42, 1145–1153. 10.1016/j.jpsychires.2008.01.010 18313696

[B169] Oyola M. G. Handa R. J. (2017). Hypothalamic-pituitary-adrenal and hypothalamic-pituitary-gonadal axes: sex differences in regulation of stress responsivity. Stress 20, 476–494. 10.1080/10253890.2017.1369523 28859530 PMC5815295

[B170] Palma-Gudiel H. Peralta V. Deuschle M. Navarro V. Fañanás L. (2019). Epigenetics-by-sex interaction for somatization conferred by methylation at the promoter region of SLC6A4 gene. Prog. Neuropsychopharmacol. Biol. Psychiatry 89, 125–131. 10.1016/j.pnpbp.2018.09.002 30201454

[B171] Pampallona S. Bollini P. Tibaldi G. Kupelnick B. Munizza C. (2004). Combined pharmacotherapy and psychological treatment for depression: a systematic review. Arch. Gen. Psychiatry 61, 714–719. 10.1001/archpsyc.61.7.714 15237083

[B172] Papale L. A. Li S. Madrid A. Zhang Q. Chen L. Chopra P.. (2016). Sex-specific hippocampal 5-hydroxymethylcytosine is disrupted in response to acute stress. Neurobiol. Dis. 96, 54–66. 10.1016/j.nbd.2016.08.014 27576189 PMC5103857

[B173] Park C. Rosenblat J. D. Brietzke E. Pan Z. Lee Y. Cao B.. (2019). Stress, epigenetics and depression: a systematic review. Neurosci. Biobehav. Rev. 102, 139–152. 10.1016/j.neubiorev.2019.04.010 31005627

[B174] Peay D. N. Saribekyan H. M. Parada P. A. Hanson E. M. Badaruddin B. S. Judd J. M.. (2020). Chronic unpredictable intermittent restraint stress disrupts spatial memory in male, but not female rats. Behav. Brain Res. 383:112519. 10.1016/j.bbr.2020.112519 32006567 PMC7059984

[B175] Philibert R. Madan A. Andersen A. Cadoret R. Packer H. Sandhu H. (2007). Serotonin transporter mRNA levels are associated with the methylation of an upstream CpG island. Am. J. Med. Genet. B Neuropsychiatr. Genet. 144B, 101–105. 10.1002/ajmg.b.30414 16958039

[B176] Pinheiro I. Dejager L. Libert C. (2011). X-chromosome-located microRNAs in immunity: might they explain male/female differences? The X chromosome-genomic context may affect X-located miRNAs and downstream signaling, thereby contributing to the enhanced immune response of females. Bioessays 33, 791–802. 10.1002/bies.201100047 21953569

[B177] Poulter M. O. Du L. Weaver I. C. Palkovits M. Faludi G. Merali Z.. (2008). GABAA receptor promoter hypermethylation in suicide brain: implications for the involvement of epigenetic processes. Biol. Psychiatry 64, 645–652. 10.1016/j.biopsych.2008.05.028 18639864

[B178] Punzi G. Ursini G. Viscanti G. Radulescu E. Shin J. H. Quarto T.. (2019). Association of a noncoding RNA postmortem with suicide by violent means and *in vivo* with aggressive phenotypes. Biol. Psychiatry 85, 417–424. 10.1016/j.biopsych.2018.11.002 30600091

[B179] Qureshi I. A. Mehler M. F. (2012). Emerging roles of non-coding RNAs in brain evolution, development, plasticity and disease. Nat. Rev. Neurosci. 13, 528–541. 10.1038/nrn3234 22814587 PMC3478095

[B180] Raison C. L. Miller A. H. (2013). The evolutionary significance of depression in Pathogen Host Defense (PATHOS-D). Mol. Psychiatry 18, 15–37. 10.1038/mp.2012.2 22290120 PMC3532038

[B181] Reinius B. Shi C. Hengshuo L. Sandhu K. S. Radomska K. J. Rosen G. D.. (2010). Female-biased expression of long non-coding RNAs in domains that escape X-inactivation in mouse. BMC Genomics 11:614. 10.1186/1471-2164-11-614 21047393 PMC3091755

[B182] Renthal W. Maze I. Krishnan V. Covington H. E. III. Xiao G. Kumar A.. (2007). Histone deacetylase 5 epigenetically controls behavioral adaptations to chronic emotional stimuli. Neuron 56, 517–529. 10.1016/j.neuron.2007.09.032 17988634

[B183] Roy B. Dunbar M. Shelton R. C. Dwivedi Y. (2017). Identification of MicroRNA-124–3p as a putative epigenetic signature of major depressive disorder. Neuropsychopharmacology 42, 864–875. 10.1038/npp.2016.175 27577603 PMC5312059

[B184] Roy B. Wang Q. Dwivedi Y. (2018). Long noncoding RNA-associated transcriptomic changes in resiliency or susceptibility to depression and response to antidepressant treatment. Int. J. Neuropsychopharmacol. 21, 461–472. 10.1111/bcpt.13595 29390069 PMC5932471

[B186] Russo S. J. Murrough J. W. Han M.-H. Charney D. S. Nestler E. J. (2012). Neurobiology of resilience. Nat. Neurosci. 15, 1475–1484. 10.1038/nn.3234 23064380 PMC3580862

[B185] Russo S. J. Nestler E. J. (2013). The brain reward circuitry in mood disorders. Nat. Rev. Neurosci. 14, 609–625. 10.1038/nrn3381 23942470 PMC3867253

[B187] Sahakyan A. Yang Y. Plath K. (2018). The role of xist in X-chromosome dosage compensation. Trends Cell Biol. 28, 999–1013. 10.1016/j.tcb.2018.05.005 29910081 PMC6249047

[B188] Sahay A. Hen R. (2007). Adult hippocampal neurogenesis in depression. Nat. Neurosci. 10, 1110–1115. 10.1038/nn1969 17726477

[B189] Sakai Y. Li H. Inaba H. Funayama Y. Ishimori E. Kawatake-Kuno A.. (2021). Gene-environment interactions mediate stress susceptibility and resilience through the CaMKIIβ/TARPγ-8/AMPAR pathway. iScience 24:102504. 10.1016/j.isci.2021.102504 34113835 PMC8170005

[B190] Sales A. J. Biojone C. Terceti M. S. Guimaraes F. S. Gomes M. V. Joca S. R. (2011). Antidepressant-like effect induced by systemic and intra-hippocampal administration of DNA methylation inhibitors. Br. J. Pharmacol. 164, 1711–1721. 10.1111/j.1476-5381.2011.01489.x 21585346 PMC3230817

[B191] Sando R. III. Gounko N. Pieraut S. Liao L. Yates J. III. Maximov A. (2012). HDAC4 governs a transcriptional program essential for synaptic plasticity and memory. Cell 151, 821–834. 10.1016/j.cell.2012.09.037 23141539 PMC3496186

[B192] Schratt G. M. Tuebing F. Nigh E. A. Kane C. G. Sabatini M. E. Kiebler M.. (2006). A brain-specific microRNA regulates dendritic spine development. Nature 439, 283–289. 10.1038/nature04367 16421561

[B193] Seki T. Yamagata H. Uchida S. Chen C. Kobayashi A. Kobayashi M.. (2019). Altered expression of long noncoding RNAs in patients with major depressive disorder. J. Psychiatr. Res. 117, 92–99. 10.1016/j.jpsychires.2019.07.004 31351391

[B195] Shen E. Y. Ahern T. H. Cheung I. Straubhaar J. Dincer A. Houston I.. (2015). Epigenetics and sex differences in the brain: a genome-wide comparison of histone-3 lysine-4 trimethylation (H3K4me3) in male and female mice. Exp. Neurol. 268, 21–29. 10.1016/j.expneurol.2014.08.006 25131640 PMC4329105

[B194] Shen E. Shulha H. Weng Z. Akbarian S. (2014). Regulation of histone H3K4 methylation in brain development and disease. Philos. Trans. R. Soc. Lond. B Biol. Sci. 369:20130514. 10.1098/rstb.2013.0514 25135975 PMC4142035

[B196] Shors T. J. Falduto J. Leuner B. (2004). The opposite effects of stress on dendritic spines in male vs. female rats are NMDA receptor-dependent. Eur. J. Neurosci. 19, 145–150. 10.1046/j.1460-9568.2003.03065.x 14750972 PMC3422870

[B197] Shulha H. P. Cheung I. Guo Y. Akbarian S. Weng Z. (2013). Coordinated cell type-specific epigenetic remodeling in prefrontal cortex begins before birth and continues into early adulthood. PLoS Genet. 9:e1003433. 10.1371/journal.pgen.1003433 23593028 PMC3623761

[B198] Siegle G. J. Carter C. S. Thase M. E. (2006). Use of FMRI to predict recovery from unipolar depression with cognitive behavior therapy. Am. J. Psychiatry 163, 735–738. 10.1176/appi.ajp.163.4.735 16585452

[B199] Simon G. E. Perlis R. H. (2010). Personalized medicine for depression: can we match patients with treatments? Am. J. Psychiatry 167, 1445–1455. 10.1176/appi.ajp.2010.09111680 20843873 PMC3723328

[B200] Soeiro-De-Souza M. G. Andreazza A. C. Carvalho A. F. Machado-Vieira R. Young L. T. Moreno R. A. (2013). Number of manic episodes is associated with elevated DNA oxidation in bipolar I disorder. Int. J. Neuropsychopharmacol. 16, 1505–1512. 10.1017/S1461145713000047 23449001

[B201] Sorrells S. F. Paredes M. F. Cebrian-Silla A. Sandoval K. Qi D. Kelley K. W.. (2018). Human hippocampal neurogenesis drops sharply in children to undetectable levels in adults. Nature 555, 377–381. 10.1038/nature25975 29513649 PMC6179355

[B202] Southwick S. M. Charney D. S. (2012). The science of resilience: implications for the prevention and treatment of depression. Science 338, 79–82. 10.1126/science.1222942 23042887

[B203] Sterrenburg L. Gaszner B. Boerrigter J. Santbergen L. Bramini M. Elliott E.. (2011). Chronic stress induces sex-specific alterations in methylation and expression of corticotropin-releasing factor gene in the rat. PLoS One 6:e28128. 10.1371/journal.pone.0028128 22132228 PMC3223222

[B204] Sugawara H. Iwamoto K. Bundo M. Ueda J. Miyauchi T. Komori A.. (2011). Hypermethylation of serotonin transporter gene in bipolar disorder detected by epigenome analysis of discordant monozygotic twins. Transl. Psychiatry 1:e24. 10.1038/tp.2011.26 22832526 PMC3309511

[B205] Sumazin P. Yang X. Chiu H. S. Chung W.-J. Iyer A. Llobet-Navas D.. (2011). An extensive microRNA-mediated network of RNA-RNA interactions regulates established oncogenic pathways in glioblastoma. Cell 147, 370–381. 10.1016/j.cell.2011.09.041 22000015 PMC3214599

[B206] Sun H. Kennedy P. J. Nestler E. J. (2013). Epigenetics of the depressed brain: role of histone acetylation and methylation. Neuropsychopharmacology 38, 124–137. 10.1038/npp.2012.73 22692567 PMC3521990

[B207] Suzuki M. M. Bird A. (2008). DNA methylation landscapes: provocative insights from epigenomics. Nat. Rev. Genet. 9, 465–476. 10.1038/nrg2341 18463664

[B208] Szulwach K. E. Li X. Li Y. Song C.-X. Wu H. Dai Q.. (2011). 5-hmC-mediated epigenetic dynamics during postnatal neurodevelopment and aging. Nat. Neurosci. 14, 1607–1616. 10.1038/nn.2959 22037496 PMC3292193

[B209] Tadić A. Müller-Engling L. Schlicht K. F. Kotsiari A. Dreimüller N. Kleimann A.. (2014). Methylation of the promoter of brain-derived neurotrophic factor exon IV and antidepressant response in major depression. Mol. Psychiatry 19, 281–283. 10.1038/mp.2013.58 23670489

[B210] Takebayashi M. Hisaoka K. Nishida A. Tsuchioka M. Miyoshi I. Kozuru T.. (2006). Decreased levels of whole blood glial cell line-derived neurotrophic factor (GDNF) in remitted patients with mood disorders. Int. J. Neuropsychopharmacol. 9, 607–612. 10.1017/S1461145705006085 16191208

[B211] Talarowska M. Berk M. Maes M. Galecki P. (2016). Autobiographical memory dysfunctions in depressive disorders. Psychiatry Clin. Neurosci. 70, 100–108. 10.1111/pcn.12370 26522618

[B212] Talebizadeh Z. Shah A. Ditacchio L. (2019). The potential role of a retrotransposed gene and a long noncoding RNA in regulating an X-linked chromatin gene (KDM5C): novel epigenetic mechanism in autism. Autism Res. 12, 1007–1021. 10.1002/aur.2116 31087518

[B213] Tanapat P. Hastings N. B. Reeves A. J. Gould E. (1999). Estrogen stimulates a transient increase in the number of new neurons in the dentate gyrus of the adult female rat. J. Neurosci. 19, 5792–5801. 10.1523/JNEUROSCI.19-14-05792.1999 10407020 PMC6783062

[B214] Thoenen H. (1995). Neurotrophins and neuronal plasticity. Science 270, 593–598. 10.1126/science.270.5236.593 7570017

[B215] Thompson P. M. Jahanshad N. Ching C. R. K. Salminen L. E. Thomopoulos S. I. Bright J.. (2020). ENIGMA and global neuroscience: a decade of large-scale studies of the brain in health and disease across more than 40 countries. Transl. Psychiatry 10:100. 10.1038/s41398-020-0705-1 32198361 PMC7083923

[B216] Tian F. Hu X.-Z. Wu X. Jiang H. Pan H. Marini A. M.. (2009). Dynamic chromatin remodeling events in hippocampal neurons are associated with NMDA receptor-mediated activation of Bdnf gene promoter 1. J. Neurochem. 109, 1375–1388. 10.1111/j.1471-4159.2009.06058.x 19476549

[B217] Tsai H.-W. Grant P. A. Rissman E. F. (2009). Sex differences in histone modifications in the neonatal mouse brain. Epigenetics 4, 47–53. 10.4161/epi.4.1.7288 19029819 PMC2667098

[B218] Tsankova N. Renthal W. Kumar A. Nestler E. J. (2007). Epigenetic regulation in psychiatric disorders. Nat. Rev. Neurosci. 8, 355–367. 10.1038/nrn2132 17453016

[B219] Tsankova N. M. Berton O. Renthal W. Kumar A. Neve R. L. Nestler E. J. (2006). Sustained hippocampal chromatin regulation in a mouse model of depression and antidepressant action. Nat. Neurosci. 9, 519–525. 10.1038/nn1659 16501568

[B220] Tseng P.-T. Lin P.-Y. Lee Y. Hung C.-F. Lung F.-W. Chen C.-S.. (2014). Age-associated decrease in global DNA methylation in patients with major depression. Neuropsychiatr. Dis. Treat. 10, 2105–2114. 10.2147/NDT.S71997 25419133 PMC4235206

[B221] Turecki G. (2014). The molecular bases of the suicidal brain. Nat. Rev. Neurosci. 15, 802–816. 10.1038/nrn3839 25354482 PMC5293539

[B223] Uchida S. Hara K. Kobayashi A. Funato H. Hobara T. Otsuki K.. (2010). Early life stress enhances behavioral vulnerability to stress through the activation of REST4-mediated gene transcription in the medial prefrontal cortex of rodents. J. Neurosci. 30, 15007–15018. 10.1523/JNEUROSCI.1436-10.2010 21068306 PMC6633839

[B224] Uchida S. Hara K. Kobayashi A. Otsuki K. Yamagata H. Hobara T.. (2011). Epigenetic status of GDNF in the ventral striatum determines susceptibility and adaptation to daily stressful events. Neuron 69, 359–372. 10.1016/j.neuron.2010.12.023 21262472

[B222] Uchida S. Shumyatsky G. P. (2018). Synaptically localized transcriptional regulators in memory formation. Neuroscience 370, 4–13. 10.1016/j.neuroscience.2017.07.023 28733211 PMC5773404

[B225] Uchida S. Yamagata H. Seki T. Watanabe Y. (2018). Epigenetic mechanisms of major depression: targeting neuronal plasticity. Psychiatry Clin. Neurosci. 72, 212–227. 10.1111/pcn.12621 29154458

[B226] Ulrich-Lai Y. M. Figueiredo H. F. Ostrander M. M. Choi D. C. Engeland W. C. Herman J. P. (2006). Chronic stress induces adrenal hyperplasia and hypertrophy in a subregion-specific manner. Am. J. Physiol. Endocrinol. Metab. 291, E965–E973. 10.1152/ajpendo.00070.2006 16772325

[B227] Vallianatos C. N. Iwase S. (2015). Disrupted intricacy of histone H3K4 methylation in neurodevelopmental disorders. Epigenomics 7, 503–519. 10.2217/epi.15.1 26077434 PMC4501478

[B228] Van Praag H. Kempermann G. Gage F. H. (2000). Neural consequences of environmental enrichment. Nat. Rev. Neurosci. 1, 191–198. 10.1038/35044558 11257907

[B229] Viau V. Meaney M. J. (1991). Variations in the hypothalamic-pituitary-adrenal response to stress during the estrous cycle in the rat. Endocrinology 129, 2503–2511. 10.1210/endo-129-5-2503 1657578

[B230] Wan Y. Liu Y. Wang X. Wu J. Liu K. Zhou J.. (2015). Identification of differential microRNAs in cerebrospinal fluid and serum of patients with major depressive disorder. PLoS One 10:e0121975. 10.1371/journal.pone.0121975 25763923 PMC4357380

[B231] Wang K. C. Chang H. Y. (2011). Molecular mechanisms of long noncoding RNAs. Mol. Cell 43, 904–914. 10.1016/j.molcel.2011.08.018 21925379 PMC3199020

[B232] Wang Q. Roy B. Dwivedi Y. (2019). Co-expression network modeling identifies key long non-coding RNA and mRNA modules in altering molecular phenotype to develop stress-induced depression in rats. Transl. Psychiatry 9:125. 10.1038/s41398-019-0448-z 30944317 PMC6447569

[B233] Webb A. Papp A. C. Curtis A. Newman L. C. Pietrzak M. Seweryn M.. (2015). RNA sequencing of transcriptomes in human brain regions: protein-coding and non-coding RNAs, isoforms and alleles. BMC Genomics 16:990. 10.1186/s12864-015-2207-8 26597164 PMC4657279

[B234] Weiser M. J. Foradori C. D. Handa R. J. (2010). Estrogen receptor β activation prevents glucocorticoid receptor-dependent effects of the central nucleus of the amygdala on behavior and neuroendocrine function. Brain Res. 1336, 78–88. 10.1016/j.brainres.2010.03.098 20381466 PMC2879480

[B235] Whiteford H. A. Degenhardt L. Rehm J. Baxter A. J. Ferrari A. J. Erskine H. E.. (2013). Global burden of disease attributable to mental and substance use disorders: findings from the Global Burden of Disease Study 2010. Lancet 382, 1575–1586. 10.1016/S0140-6736(13)61611-6 23993280

[B236] Yamagata H. Ogihara H. Matsuo K. Uchida S. Kobayashi A. Seki T.. (2021). Distinct epigenetic signatures between adult-onset and late-onset depression. Sci. Rep. 11:2296. 10.1038/s41598-021-81758-8 33504850 PMC7840753

[B237] Yim Y. Y. Teague C. D. Nestler E. J. (2020). *in vivo* locus-specific editing of the neuroepigenome. Nat. Rev. Neurosci. 21, 471–484. 10.1038/s41583-020-0334-y 32704051 PMC7439525

[B238] Yoshino Y. Dwivedi Y. (2020). Non-coding RNAs in psychiatric disorders and suicidal behavior. Front. Psychiatry 11:543893. 10.3389/fpsyt.2020.543893 33101077 PMC7522197

[B239] Yoshino Y. Roy B. Dwivedi Y. (2021). Differential and unique patterns of synaptic miRNA expression in dorsolateral prefrontal cortex of depressed subjects. Neuropsychopharmacology 46, 900–910. 10.1038/s41386-020-00861-y 32919404 PMC8115313

[B240] Zeng Y. Navarro P. Shirali M. Howard D. M. Adams M. J. Hall L. S.. (2017). Genome-wide regional heritability mapping identifies a locus within the TOX2 gene associated with major depressive disorder. Biol. Psychiatry 82, 312–321. 10.1016/j.biopsych.2016.12.012 28153336 PMC5553996

[B241] Zhang H.-P. Liu X.-L. Chen J.-J. Cheng K. Bai S.-J. Zheng P.. (2020). Circulating microRNA 134 sheds light on the diagnosis of major depressive disorder. Transl. Psychiatry 10:95. 10.1038/s41398-020-0773-2 32179735 PMC7075934

[B242] Zhang L. Rubinow D. R. Xaing G. Li B. S. Chang Y. H. Maric D.. (2001). Estrogen protects against β-amyloid-induced neurotoxicity in rat hippocampal neurons by activation of Akt. Neuroreport 12, 1919–1923. 10.1097/00001756-200107030-00030 11435923

[B243] Zhang Y. Zhu X. Bai M. Zhang L. Xue L. Yi J. (2013). Maternal deprivation enhances behavioral vulnerability to stress associated with miR-504 expression in nucleus accumbens of rats. PLoS One 8:e69934. 10.1371/journal.pone.0069934 23922862 PMC3724734

[B244] Zhao Y. Li H. Fang S. Kang Y. Wu W. Hao Y.. (2016). NONCODE 2016: an informative and valuable data source of long non-coding RNAs. Nucleic Acids Res. 44, D203–D208. 10.1093/nar/gkv1252 26586799 PMC4702886

[B245] Zhou Y. Lutz P.-E. Wang Y. C. Ragoussis J. Turecki G. (2018). Global long non-coding RNA expression in the rostral anterior cingulate cortex of depressed suicides. Transl. Psychiatry 8:224. 10.1038/s41398-018-0267-7 30337518 PMC6193959

[B246] Zuloaga D. G. Heck A. L. De Guzman R. M. Handa R. J. (2020). Roles for androgens in mediating the sex differences of neuroendocrine and behavioral stress responses. Biol. Sex Differ. 11:44. 10.1186/s13293-020-00319-2 32727567 PMC7388454

